# An Integrated Framework Advancing Membrane Protein Modeling and Design

**DOI:** 10.1371/journal.pcbi.1004398

**Published:** 2015-09-01

**Authors:** Rebecca F. Alford, Julia Koehler Leman, Brian D. Weitzner, Amanda M. Duran, Drew C. Tilley, Assaf Elazar, Jeffrey J. Gray

**Affiliations:** 1 Department of Chemical and Biomolecular Engineering, Johns Hopkins University, Baltimore, Maryland, United States of America; 2 Department of Chemistry, Carnegie Mellon University, Pittsburgh, Pennsylvania, United States of America; 3 Center for Structural Biology, Department of Chemistry, Vanderbilt University, Nashville, Tennessee, United States of America; 4 Department of Physiology and Membrane Biology, University of California, Davis, Davis, California, United States of America; 5 Department of Biological Chemistry, Weizmann Institute of Science, Rehovot, Israel; UNC Charlotte, UNITED STATES

## Abstract

Membrane proteins are critical functional molecules in the human body, constituting more than 30% of open reading frames in the human genome. Unfortunately, a myriad of difficulties in overexpression and reconstitution into membrane mimetics severely limit our ability to determine their structures. Computational tools are therefore instrumental to membrane protein structure prediction, consequently increasing our understanding of membrane protein function and their role in disease. Here, we describe a general framework facilitating membrane protein modeling and design that combines the scientific principles for membrane protein modeling with the flexible software architecture of Rosetta3. This new framework, called RosettaMP, provides a general membrane representation that interfaces with scoring, conformational sampling, and mutation routines that can be easily combined to create new protocols. To demonstrate the capabilities of this implementation, we developed four proof-of-concept applications for (1) prediction of free energy changes upon mutation; (2) high-resolution structural refinement; (3) protein-protein docking; and (4) assembly of symmetric protein complexes, all in the membrane environment. Preliminary data show that these algorithms can produce meaningful scores and structures. The data also suggest needed improvements to both sampling routines and score functions. Importantly, the applications collectively demonstrate the potential of combining the flexible nature of RosettaMP with the power of Rosetta algorithms to facilitate membrane protein modeling and design.

## Author Summary

Over 30% of the human proteome consists of proteins embedded in biological membranes. These proteins are critical in many processes such as transport of materials in and out of the cell and transmitting signals to other cells in the body. They are implicated in a large number of diseases; in fact, they are targeted by over 50% of pharmaceutical drugs on the market. Since the membrane environment makes experimental structure determination extremely difficult, there is a need for alternative, computational approaches. Here, we describe a new framework, RosettaMP, for computational modeling and design of membrane protein structures, integrated in the Rosetta3 software suite. This framework includes a set of tools for representing the membrane bilayer, moving the protein, altering its sequence, and estimating free energies. We demonstrate tools to predict the effects of mutations, refine atomic details of protein structures, simulate protein binding, and assemble symmetric complexes, all in the membrane bilayer. Taken together, these applications demonstrate the potential of RosettaMP to facilitate membrane protein structure prediction and design, enabling us to understand the function of these proteins and their role in human disease.

This is a *PLOS Computational Biology* Methods paper.

## Introduction

Membrane proteins are critical participants in a wide variety of biological processes including cell adhesion, signaling, transport, and enzymatic activity [1]. They comprise more than 30% of open reading frames [2] and are targeted by over half of currently available pharmaceutical drugs [3,4]. Despite their importance, our knowledge of membrane protein structure and function remains severely limited, as shown by a constant 1–2% representation of structures in the Protein Data Bank [5] over the past decade [6]. The paucity of experimentally determined structures can be attributed to wide-ranging challenges in overexpression, reconstitution into membrane mimetics, and ultimately structure determination by various methods [7]. Due to these experimental challenges, computational approaches assume a pivotal role in advancing our understanding of membrane protein structure and function.

Compared to modeling soluble proteins, membrane protein modeling has the advantage of constraining the conformational search space into the two dimensions of the membrane bilayer, which imposes structural constraints onto the protein. Whereas soluble proteins exhibit enormous structural diversity, the structural motifs in the membrane environment are either α-helical bundles or β-barrels. Since these folds are formed by secondary structure elements adopting preferred orientations in the ordered environment of the lipid bilayer, the use of adapted sampling techniques could substantially increase conformational sampling efficiency. A higher sampling efficiency is required because membrane proteins are typically much larger in size, offsetting the reduction in conformational search space.

Additionally, computational methods for membrane protein modeling require reliable free energy calculations or score functions to distinguish native-like from non-native conformations. Therefore, an accurate representation of the heterogeneous environment of the lipid bilayer is needed. The membrane bilayer can be represented implicitly by using a layered, continuum solvation model, which is computationally inexpensive but unable to describe membrane fluctuations or specific membrane protein—lipid interactions. An additional challenge for the score function is that the precise location of the lipid bilayer surrounding the protein in experimental structures is unknown because the membrane mimetic evades experimental observation.

The aforementioned challenges and the lack of experimental structures have delayed the development and therefore availability of high-quality computational methods for membrane protein modeling, compared to available methods for soluble protein modeling. Whereas soluble protein modeling increasingly focuses on high-resolution structural features as in docking, design and ligand docking applications, methods for membrane protein modeling still mainly focus on obtaining models for unknown protein structures.

Four main techniques for computational modeling of membrane proteins are available: (1) Since template structures for homology modeling are unavailable for many membrane proteins of interest, ***ab initio* modeling** is an important technique (*e*.*g*. using BCL∷MPFold [8–15]). *Ab initio* structure prediction is one of the most difficult of the modeling tasks, yet it also has the largest benefits because of its ability to predict novel folds. Additionally, in contrast to homology modeling where the final model can contain artifacts from the template, models from *ab initio* structure prediction are not biased by previously determined protein structures. (2) For low (~25%) to very low (~5%) sequence similarities to a known structure, **fold recognition** techniques generate a low-resolution protein model; the accuracy of these models rarely achieves better than 3–4 Å RMSD. (3) **Homology modeling** can be used to model the three-dimensional structure of a query protein if the sequence similarity between the query sequence and the sequence of a template structure is greater than ~30%. The recent increase in determined membrane protein structures (and therefore template availability) has elevated the quality and number of built homology models. Recently, GPCR homology models with an RMSD as low as 2.9 Å from the target structure were created from starting templates with a sequence identity as low as 15% [16]. (4) If the structure of the membrane protein is known, **molecular dynamics (MD) simulations** can follow time trajectories of proteins and lipids in full-atom representation with physics-based energy functions to investigate high-resolution phenomena such as ion channel gating or transport across the membrane [17–19]. With the recent increase in available membrane protein structures, high-resolution modeling methods including **protein design** have started to emerge [20–25]. Two notable achievements include a helix—helix interface design [21] and a design of a four-helix bundle that selectively transports metal ions across the membrane [20].

A limitation of many membrane protein modeling tools is high specialization to accomplish a single task; thus these methods are not easily combined with other modeling tools. The membrane protein community would benefit from an integrated tool that is able to carry out a variety of complex modeling tasks such as loop modeling, predicting the effects of mutations, design, docking, symmetric complex assembly, and ligand docking, in addition to *ab initio* structure prediction, homology modeling, and high-resolution refinement. Additionally, integrated methods would enable testing of a score function in multiple contexts to more rapidly converge on a universal score function in the bilayer environment. The Rosetta software suite offers an integrated toolset for biomolecular modeling, docking, and design, including a broadly tested and refined score function for soluble biomolecules. Moreover, Rosetta has two pioneering membrane protein modeling applications, RosettaMembrane *ab initio* [26] and relax [23].

The RosettaMembrane *ab initio* protocol [23,26] was one of the first methods for *ab initio* structure prediction of membrane proteins. It combines Rosetta’s *ab initio* structure prediction protocol for soluble proteins [27] with a low-resolution score function derived from a database of structures of membrane proteins [26,28,29]. This method was later updated to include a high-resolution refinement stage [23,30] that uses an all-atom score function based on the Lazaridis implicit Gaussian-exclusion solvation model for atoms in the membrane [31]. Recently, RosettaMembrane was also used to model transmembrane helical proteins from distant homologues [16].

Since the creation of the RosettaMembrane *ab initio* protocol in 2006, Rosetta has been reorganized into a set of object-oriented libraries (“Rosetta3”) while RosettaMembrane remained in its original implementation. Rosetta3 is now a cohesive, flexible software suite that includes separate objects for conformation and scoring, interaction graphs, score functions organized by multi-body dependencies, kinematics managed through a fold tree, maps to identify flexible portions of the molecule(s), job distribution, and scripting interfaces [32]. Continuous improvements and additions to Rosetta’s extensive library of complex tools motivate its use for membrane protein modeling. However, the scientific concepts of the original RosettaMembrane require integration and generalization to be compatible with the object-oriented architecture of Rosetta3.

Here, we present a new framework, called RosettaMP, integrated in the Rosetta software suite, which enables the development of novel protocols for membrane protein modeling and design. We describe the new, central building blocks to represent the membrane bilayer, and to sample and score both conformations and sequences. We used RosettaMP to create four proof-of-concept applications: (1) prediction of free energy changes upon mutation, (2) high-resolution structural refinement, (3) protein—protein docking, and (4) assembly of symmetric complexes, all in the membrane bilayer. The protocols can be accessed via command line, PyRosetta [33], and RosettaScripts [34], with various levels of customizability for both developers and users. Using a set of test cases, we are able to obtain information on the applicability of the existing score function in these wider contexts. Collectively, the applications demonstrate how RosettaMP and existing Rosetta protocols can be combined to quickly create powerful new methods to answer a broad range of scientific questions. Because of its ability to interoperate with existing Rosetta code and reusable representation of the lipid bilayer, RosettaMP substantially lowers the barrier in complexity for the development of new protocols to model and design membrane proteins, opening the door to many new, critically needed methods.

## Methods

### Design of RosettaMP

#### RosettaMP extends the object-oriented architecture of Rosetta3

Our goal was to create a flexible software architecture for modeling membrane proteins; we therefore used object-oriented design principles [35] to encapsulate individual scientific concepts for membrane protein modeling into a set of software ‘building blocks’, or objects. Each object includes information (data) and actions (methods) required to represent a given scientific concept. Together, well-designed objects interact within the larger infrastructure to perform specific tasks.

**Fig 1:**
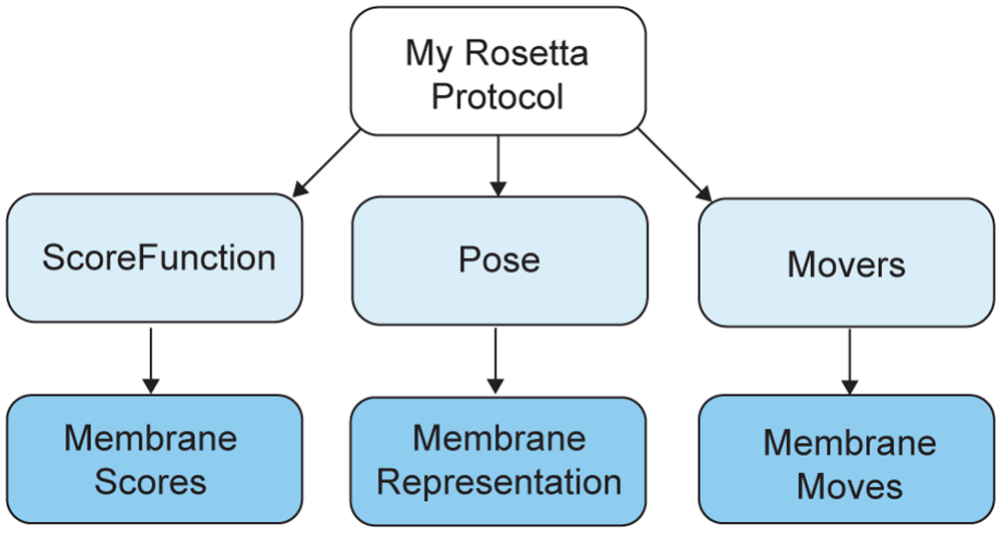
RosettaMP directly extends the architecture of Rosetta3. Every Rosetta protocol requires at least these three main objects for modeling or design tasks (light blue): a Pose to a represent a biomolecule, a ScoreFunction to rank modeled structures and sequences, and Movers to sample new conformations of the Pose. RosettaMP directly extends this architecture (blue) by adding an element to the Pose representing the membrane bilayer, restructuring the original membrane ScoreFunction to rely on this membrane representation, and implementing a new set of Movers to sample the conformational search space available in the membrane bilayer.

A Rosetta simulation is centered on one or more biomolecules (protein, protein-ligand complex, nucleic acids, etc.), which are stored in an object called a Pose. Sampling in the conformational search space is accomplished by manipulating the Pose by Movers and evaluating the resulting conformations using a ScoreFunction [32]. We expanded this infrastructure by creating new objects to represent the membrane environment and enable scoring and sampling routines that account for the lipid bilayer (Fig 1). Data that describes the membrane, such as membrane location and thickness, is stored in an object encapsulated in the Pose. Membrane-specific Movers sample the conformational search space exploiting the implicit constraints imposed by the membrane bilayer. Membrane-specific ScoreFunction terms use the geometrical information provided by the Pose. This code design separates conformation and score evaluation, and preserves the score models of the original membrane score functions.

#### Membrane data is stored centrally in the Pose

**Fig 2:**
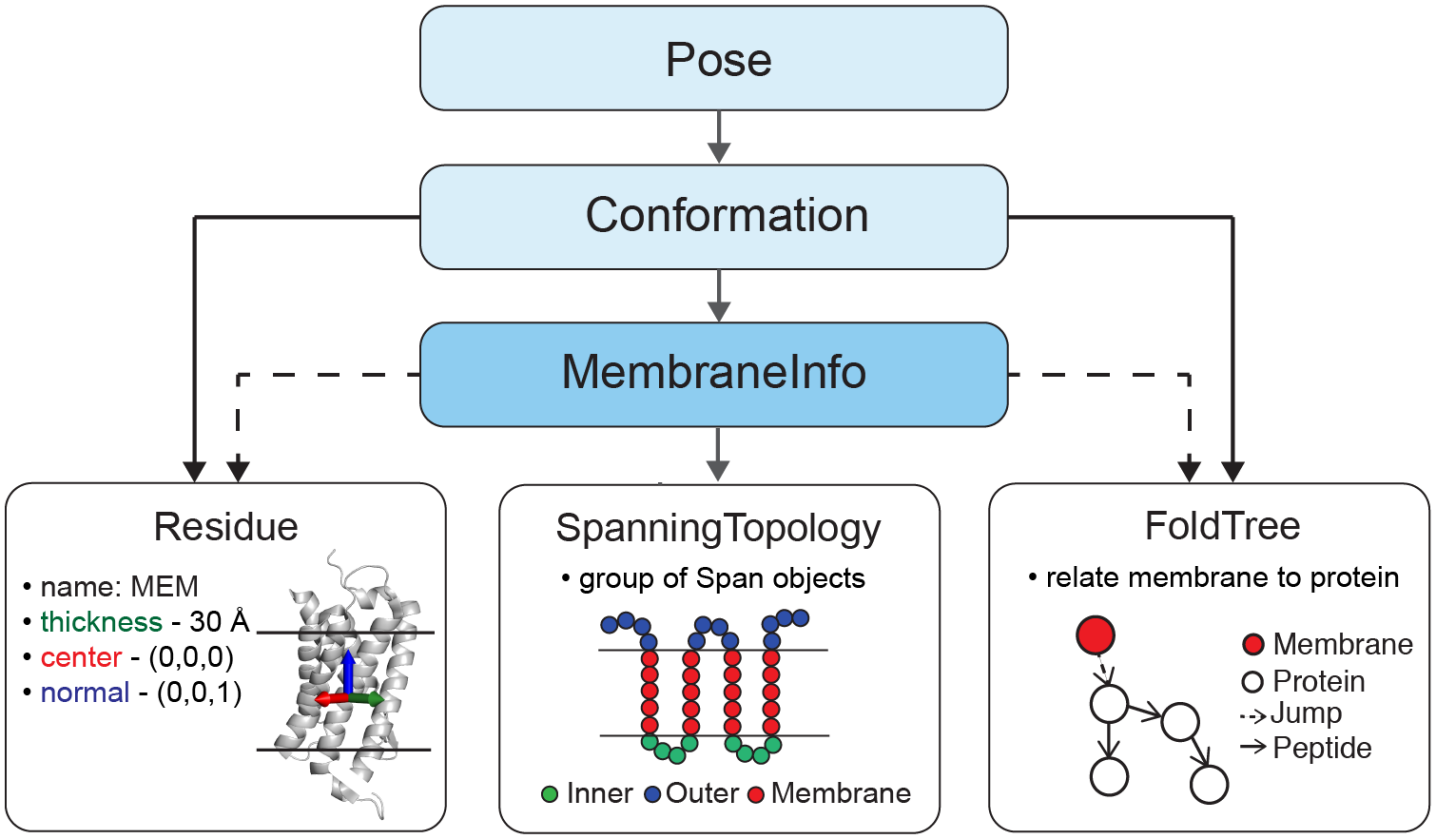
Detailed architecture of RosettaMP. RosettaMP represents the membrane bilayer using three main components connected to a central **MembraneInfo** object (blue). MembraneInfo stores information needed to represent the membrane (line arrows) and tracks information present in the Pose (dotted arrows). A special **Residue** type is added to the Pose, describing the geometry of the membrane bilayer by coordinates storing the center, normal and thickness of the bilayer. A **SpanningTopology** object describes the transmembrane regions of the Pose. The **FoldTree** uses a jump edge to establish the connection between the membrane residue and the protein. MembraneInfo is also a central repository for membrane-related features such as lipid accessibility of each residue (LipidAccInfo). A full Universal Markup Language (UML) diagram is presented in Fig A in S1 File.

To extend Rosetta’s description of a biomolecule to include the lipid bilayer, we created a class called MembraneInfo to store all information necessary for representing a membrane Pose. MembraneInfo attaches a virtual residue to the Pose (see below and Fig B in S1 File) that represents the membrane bilayer chemistry and geometry such as its center, normal and thickness. MembraneInfo also stores descriptors of sequence- and structure-based membrane protein properties, such as transmembrane spans. MembraneInfo is a member of the Conformation object, which is part of the Pose (Fig 2 and Fig A and Table A in S1 File). Because the Pose is the central object in Rosetta protocols, the information in MembraneInfo is readily accessible through the Pose, enabling access to the membrane representation in any Rosetta protocol.

#### Membrane position and thickness is stored in a Residue object

Molecules in Rosetta are represented as a set of Residue objects. For proteins or nucleic acids, the data structure corresponds to the chemical residue (e.g., an alanine or cytosine), but in Rosetta a Residue can be used more generally to encapsulate a set of related coordinates. Thus, to represent the membrane ‘conformation,’ we use a ‘Residue’ object with three ‘virtual’ atoms that define the membrane center, normal vector, and thickness. As a Residue, the membrane can be fixed or moveable during modeling using the same machinery as with the biomolecular residues. The default membrane thickness is 30 Å, including both the hydrophobic membrane core and the membrane-water interfacial regions.

#### Connection between the protein and membrane is established through the FoldTree

To allow either the membrane or the protein to be fixed or moveable in the coordinate frame, we use Rosetta’s FoldTree object. As described previously [36], the FoldTree is a rooted, directed, acyclic graph that describes the chemical and geometric connectivity of the Residues in the Pose. Since Rosetta uses an internal coordinate system, atomic coordinates are described relative to each other by using distances, angles, and dihedral angles for covalently bonded atoms, or rigid body transformations for non-covalently-bonded atoms. The FoldTree defines the order of residues (i.e. connections) in which the coordinate update takes place after a conformational sampling step. Coordinates are updated starting at a *root* and propagate along the connections in the FoldTree, called Edges. These connections can be peptide bonds, called *peptide edges*, or non-bonding connections, called *jump edges*. Jump edges in the FoldTree therefore maintain long-range connections, such as interactions between a protein and a ligand, positions of anchor residues for loop building, or relative positions of domains during the assembly of multi-domain proteins.

In RosettaMP, we use a jump edge in the FoldTree to connect the membrane residue to the protein. Adjusting the position of the root in the FoldTree will subsequently invert jump and peptide edges (i.e. coordinate update takes place in the opposite direction) and therefore changes which parts of the pose are fixed or movable. For instance, if the membrane residue is at the root of the FoldTree, it will stay fixed, whereas the protein will move in the coordinate system of the membrane. In contrast, if the root is a residue in the protein, this residue will stay fixed and the membrane residue will move in the coordinate system of the protein.

#### Membrane protein topology is stored in the SpanningTopology object

*Ab initio* structure prediction for membrane proteins often begins with an estimate of the number and location of the transmembrane spans of secondary structure. Transmembrane spans can be predicted from the protein sequence using servers such as OCTOPUS [37] or BCL::Jufo9D [38]. In RosettaMP, the start and end residue numbers of a single transmembrane span are stored in a Span object. All Span objects for the protein are gathered in a SpanningTopology container. If an experimental protein structure is available, the span_from_pdb application (S7 File) can be used to create a span file for RosettaMP protocols. The protein structure must be transformed into the membrane coordinate frame, *e*.*g*. by using the PDBTM [39] or OPM [40] databases or the TMDET [41] or PPM [42] servers.

#### RosettaMembrane score functions were restructured to use the new framework

The original implementation of RosettaMembrane includes a low-resolution and high-resolution score function [23,26,43]. We restructured both score functions to follow Rosetta3’s decomposition of energies into one-body, two-body, and whole protein terms [32]. The new implementation uses the membrane position defined in the membrane residue to score per-residue and residue pair interactions within the hydrophobic layers. The scientific integrity of the restructured membrane score function code was verified using continuous regression testing against the original implementation. The new implementation is now compatible with a fixed or a movable membrane and enables facile adjustment of the score functions. The score function terms are explained in Table 1 with formulas in Table B in S1 File and weights in Tables C-D in S1 File.

**Table 1:** Rosetta membrane energy terms used by RosettaMP.

Term	Resolution	Scope^1^	Description	Ref
mp_env	low	1b, z, cd	Knowledge-based potential describing propensity for a single residue to be at a given depth in the membrane and burial by residues	[26]
mp_pair	low	2b, z	Knowledge-based pairwise interaction potential between two residues some distance apart at a given depth in the membrane	[26]
mp_cbeta	low	1b, cd	Knowledge-based residue density potential based on number of neighbor residues and conditional upon number of transmembrane helices	[26,28]
mp_lipo	low	1b	Scores agreement between predicted lipophilicity (from LIPS server) and the model	[29]
mp_nonhelix	low	1b, z	Penalty for non-helical secondary structure in the membrane	[26]
mp_termini	low	1b, z	Penalty for residues outside of the hydrophobic layer of the membrane	[26]
mp_tmproj	low	ws	Penalty for transmembrane helices that project outside of the membrane	[26]
fa_mpenv	high	1b, z	Free energy of a single, isolated atom in solvent or lipid, depending on the depth in the membrane	[23,31]
fa_mpsolv	high	2b, z, cd	Atomic solvation free energy change due to the presence of surrounding atoms, modeled via Gaussian exclusion	[23,31]
fa_mpenv_ smooth	high	1b, z, cd	Knowledge-based potential describing propensity for a single atom to be at a given depth in the membrane	[43]
hbond	high	2b, z	Depth-adjusted Rosetta hydrogen bonding term with stronger hydrogen bonding in the membrane	[23,30]

^1^ Scope of individual energy terms. 1b indicates a per-residue or per-atom score (one-body), 2b indicates a two-body score, z indicates the score is dependent upon depth in the membrane bilayer, cd indicates the score depends on context (typically the number of surrounding residues), and ws is a score based on the whole structure.

#### Specific Movers invoke RosettaMP and position the protein in the membrane

In Rosetta3 protocols, Movers are used to manipulate the Pose by conformation or sequence sampling. In RosettaMP, we use Movers to setup required membrane information and manipulate conformations in accordance with the implicit constraints the membrane imposes on the structure of these proteins.

The Mover that invokes RosettaMP and instantiates a membrane Pose is the **AddMembraneMover**. The AddMembraneMover adds the membrane residue to the Pose, sets up a default FoldTree, and initializes MembraneInfo. The position of the root in the FoldTree for either a fixed membrane/movable protein or movable membrane/fixed protein is determined by the specific protocol.

The **SetMembranePositionMover** sets the center and normal of the membrane representation to pre-computed values for a fixed protein. These values can be computed from PPM [42] or TMDET [41], two servers that transform membrane protein structures into membrane coordinates with the membrane center at *z* = 0 and membrane normal along the *z*-axis.

The **MembranePositionFromTopologyMover** uses knowledge of the transmembrane spans (stored in the SpanningTopology object) and protein coordinates to compute the centers and normals of each transmembrane span, average them, and then set the membrane center and normal to these values. Thus, this mover provides a first estimation that can be subsequently refined (as for instance in the MPrelax application below).

Similar to the previous mover, the **TransformIntoMembraneMover** uses the transmembrane spans and the protein coordinates to compute the centers and normal of each transmembrane span, and averages those to estimate an initial membrane position for the whole protein. This Mover then transforms the protein into membrane coordinates such that the centers and normals of the overall estimated values and the membrane residue coincide [default center at (0, 0, 0) and normal at (0, 0, 15), where 15 Å represents half of the bilayer thickness].

#### Applications can invoke real-time visualization of a protocol in PyMOL

Visualizing the membrane bilayer is useful for evaluating the protein position in the membrane during the simulation and for final models. Rosetta’s PyMOLMover transmits coordinate information from the Pose in Rosetta to PyMOL in real time [44]. We extended this mover to additionally transfer membrane information, allowing the membrane bilayer to be displayed in PyMOL [45] when RosettaMP is in use (Fig J in S1 File). The PyMOLMover extracts the center, normal and thickness of the membrane bilayer from the membrane residue and uses this information to compute the location of two parallel planes representing the membrane. The planes are drawn as compiled graphics objects (CGO) [46] and are automatically updated after a change in position or orientation of the bilayer during a simulation. The visualization is demonstrated in the protocol capture in S6 File and S1 Movie.

## Results

### RosettaMP Applications

#### Creating new membrane protein modeling and design applications is simplified

To demonstrate the flexibility and potential of RosettaMP, we developed four proof-of-concept applications that draw on the rich existing functionality of Rosetta3 and adapt them for membrane proteins. The four applications are the prediction of free energy changes upon mutation (MPddG), high-resolution structural refinement (MPrelax), protein-protein docking (MPdock), and assembly of symmetric complexes (MPsymdock; Table 2). These protocols demonstrate use of mutations (design), energetic optimization, rigid-body transformations, and use of the symmetry machinery, all in the membrane environment. They further use three interfaces with the Rosetta libraries: writing code in C++ and scripting in PyRosetta [33] and RosettaScripts [34].

**Table 2:** Applications developed with RosettaMP.

Application	Description	Platform
MPddG	Prediction of ΔΔG of mutation in the membrane	PyRosetta
MPrelax	High-resolution refinement in the membrane	RosettaScripts
MPdock	Protein-protein docking in the membrane	Rosetta3
MPsymdock	Assembly of symmetric complexes in the membrane	Rosetta3

**Fig 3:**
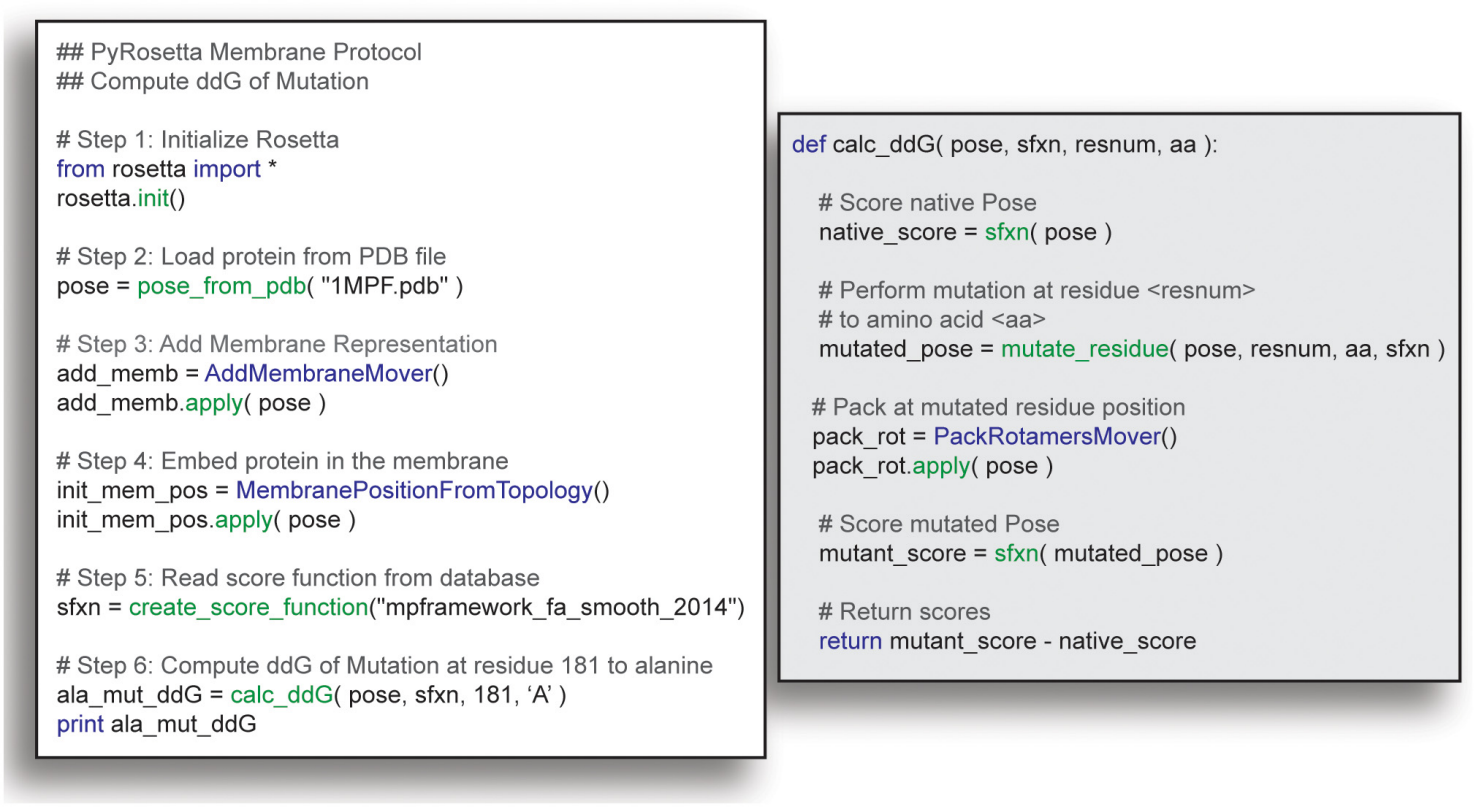
PyRosetta script for calculating the ΔΔ*G* of mutation via RosettaMP. This example script loads Rosetta, adds the membrane representation, and uses the membrane score function to compute the ΔΔ*G* of mutation in the membrane. Left: Python script used for ΔΔ*G* calculations. Right: calc_ddG method used for computing ΔΔ*G* of mutation.

In Fig 3, we illustrate the simplicity of combining RosettaMP with existing Rosetta functions to create a useful application. The PyRosetta script consists of six steps: initializing Rosetta, loading a protein, adding a membrane, positioning the membrane using a predicted spanning topology, initializing a score function, and then calling PyRosetta’s ΔΔ*G* calculation, a simplified version of a ΔΔ*G* calculation protocol described below. This ΔΔ*G* calculation simply optimizes side chain conformations and scores the protein with the wild-type and mutant residue at a given position.

#### Predicting free-energy changes (ΔΔG) upon mutation in the membrane (MPddG)

Measuring the thermodynamic cost of a mutation provides insights into protein stability and serves as the starting point for protein design. Rosetta has thus far been used to estimate the free energy changes upon mutation (ΔΔ*G*) for proteins in solution [47] and for interfaces between soluble proteins [48,49]. To predict the ΔΔ*G* of mutation in the membrane environment (protocol capture in S2 File), we combined RosettaMP with a fixed backbone ΔΔ*G* prediction protocol, similar to the one described in Kellogg *et al*. [47]. After mutation, side chain conformations were optimized for all residues within 8 Å of the mutated residue, and the ΔΔ*G* was computed as the difference in Rosetta Energy Units (REU) between the mutant and native structure.

**Fig 4:**
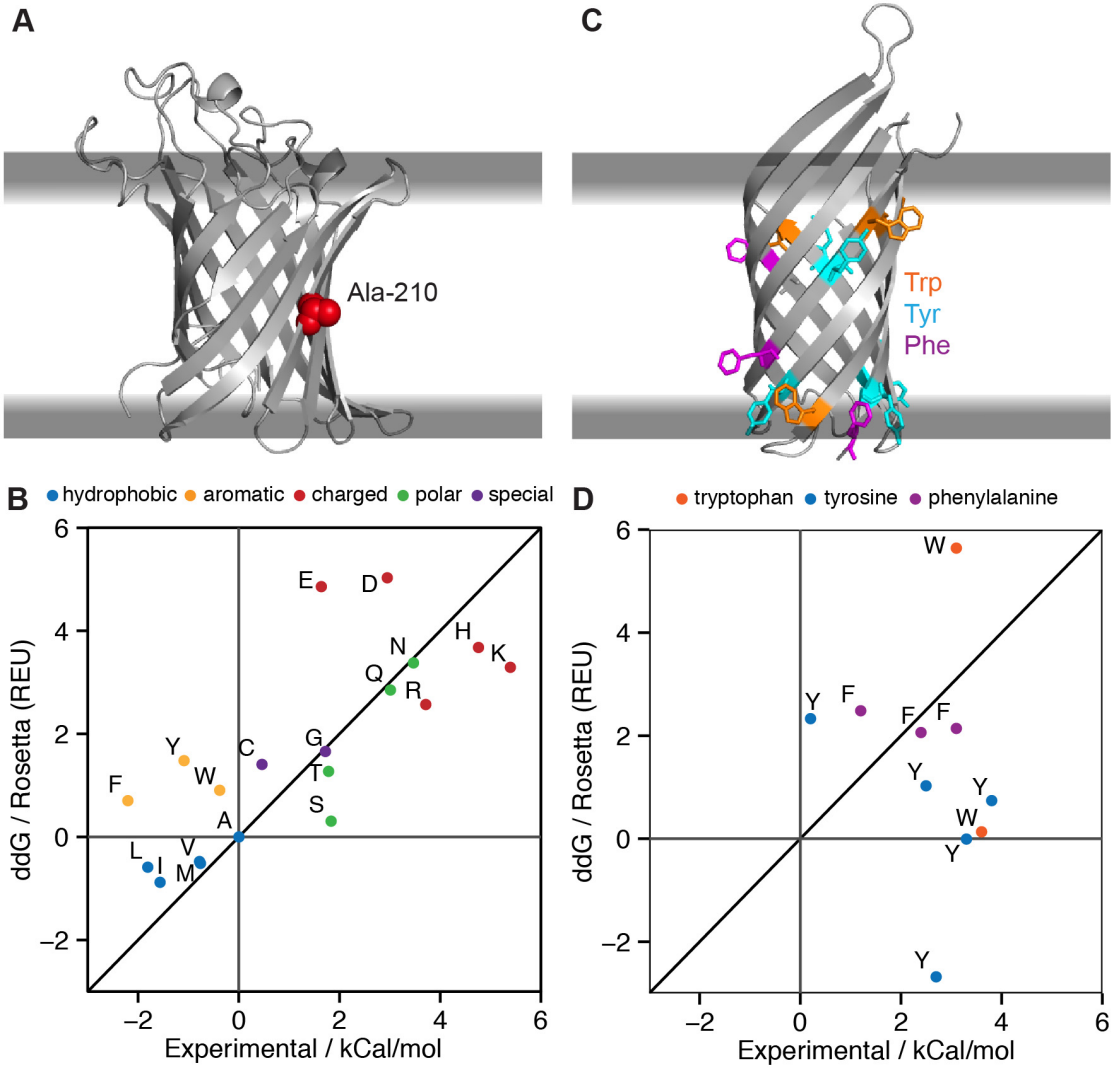
MPddG computes free energy changes upon mutation in the membrane environment (ΔΔG). (A) Outer membrane protein phospholipase A (OmpLA, PDB 1qd6) with its native alanine at position 210 in red at the center of the membrane. (B) Plot of RosettaMP-calculated fixed-backbone ΔΔ*G*s versus experimentally measured values of Moon & Fleming for variants at position 210 [50]. Proline is off-scale (ΔΔ*G*_*pred*_ = 193.2 REU) due to incompatible backbone torsions yielding ring closure penalties. (C) Outer membrane protein A (OmpA, PDB 1qjp) with aromatic residues mutated to alanine at various interfacial positions. (D) Plot of RosettaMP-calculated ΔΔ*G*s versus experimentally measured values of Hong & Tamm [51]. The mutation W15A is off-scale (ΔΔ*G*_pred_ = -43.0 REU) due to the loss of repulsive interactions upon mutation to alanine. Both (B) and (D) include a line for *y* = *x*.

We tested the ΔΔ*G* application’s ability to reproduce experimental ΔΔ*G* values from two data sets: (1) comprehensive mutations on outer membrane protein phospholipase A (OmpLA) [50] at a single position at the center of the membrane (Fig 4A), and (2) mutations from aromatic residues to alanine at several interfacial positions (Fig 4C) in outer membrane protein A (OmpA) [51]. Calculated values are compared to the experimental measurements in Fig 4B and 4D. A complete breakdown of predicted ΔΔ*G* values by Rosetta score term is shown in Tables E-F in S1 File.

**Fig 5:**
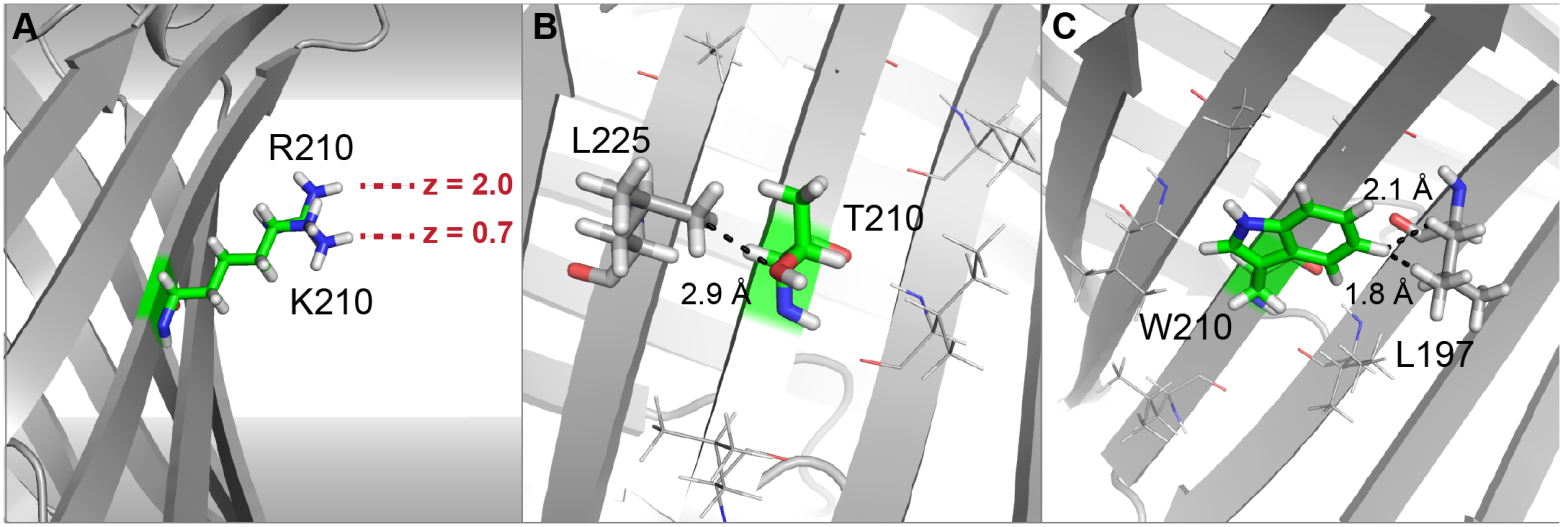
Structures of mutant residues at position 210 of OmpLA. (A) The charged residues arginine and lysine (superimposed) cannot reach the interface region. The *z*-coordinate shows the difference in membrane depth of the two charged side chains. Membrane environment scores are unfavorable for both, with lysine being slightly more unfavorable. (B) Insertion of threonine at position 210 is penalized by a mild clash from the neighboring leucine 225; serine at this position is accommodated more easily (Fig D in S1 File). (C) The tryptophan side chain is close to the neighboring leucine 197, resulting in large repulsive scores. All aromatic mutations have a comparably large repulsive van der Waals and rotamer scores, resulting in over-prediction of their ΔΔ*G* values.

For OmpLA, an alanine at the center of the membrane is mutated into all 19 other amino acids. Insertion of proline is significantly overestimated because unfavorable dihedral angles cause a kink in the β-strand that cannot be resolved due to the fixed-backbone assumption. The negatively charged residues aspartic acid and glutamic acid are also over-predicted, similar to comparisons of these experimental values with published hydrophobicity scales [50]. When aspartic acid, glutamic acid, and proline are excluded, the correlation between the experimental and calculated values is *R* = 0.86, and the calculated Rosetta Energy Units (REU) correspond roughly to the measurements in kcal/mol. RosettaMP predicts the insertion of an arginine at the center of the membrane to be less disruptive than insertion of a lysine, and the side chain stretches toward the membrane interface (Fig 5A). This result matches previous experimental values [50,52,53] and occurs because the longer, positively charged side chain of arginine can snorkel further towards the interface region (Fig C in S1 File) and interact with charged lipid head groups or interfacial water molecules [54]. The preference for arginine over lysine arises from the ~0.7 REU difference in the environment score (fa_mpenv_smooth) while small variations in the other score terms balance out. ΔΔ*G* values for the polar residues asparagine and glutamic acid are consistent with the published values [50].

The Lazaridis membrane environment energy (fa_mpenv) correctly identifies both threonine and serine to be unfavorable, yet the ΔΔ*G* value of serine is under-predicted. The ΔΔ*G* of threonine is calculated to be greater than for serine due to larger van der Waals repulsive scores from a clash with leucine 225 (Fig 5B and Fig D in S1 File). Mutations into aromatic residues (phenylalanine, tryptophan and tyrosine) are calculated to be disruptive, which is in agreement with other hydrophobicity scales [55–57] but disagrees with published values [50]. In the RosettaMP calculations, the positive ΔΔ*G* mainly arises from unfavorable van der Waals repulsive energy (fa_rep) (Fig 5C) and the rotamer score (fa_dun) due to clashes with leucine 225 (Fig E in S1 File). Insertion of negatively charged residues, aspartic acid and glutamic acid, is significantly overestimated, accounting for the cost of placing a charged species into the membrane, which should add about 2 kcal/mol [50]. These findings suggest that further improvements to the score function are necessary.

Our second data set is from OmpA. The data points cluster from calculated values of 0–3 REU and experimental values 0–4 kcal/mol, capturing disruption of π-orbital stacking interactions with neighboring aromatic residues, as shown by large changes in the van der Waals attractive scores. Although the correlation between predictions and published values is low (*R* = 0.13), the data in Fig 4D demonstrate that the MPddG application correctly identifies 9 of 12 mutations as unfavorable, attributed to loss of van der Waals attractive energy and an unfavorable knowledge-based membrane environment score (Table F in S1 File). The two tyrosine residues and single tryptophan whose mutation to alanine are predicted to be favorable (ΔΔ*G*_pred_ < 0) are isolated residues without any π-stacking interactions that would be disrupted by the mutation. In each case, alanine is predicted to be more stable because the unfavorable van der Waals attractive score difference is outweighed by the sum of the favorable repulsive and rotamer score differences. To achieve high-quality predictions of ΔΔ*G*s of mutation, possible improvements include consideration of backbone and side chain flexibility [47] and advances in the score function, both of which require extensive benchmarking on a large dataset, which is currently difficult to obtain. Therefore, identification of favorable vs. disruptive mutations is an important first step towards this goal.

#### High-resolution refinement of membrane protein structures (MPrelax)

High-resolution refinement of protein structures is necessary to advance low-resolution structures to atomic level detail and to create high-resolution models as inputs for protein-protein docking, protein-ligand docking, and design. For membrane proteins, this task is complicated by lack of knowing the precise embedding of the protein in the membrane.

**Fig 6:**
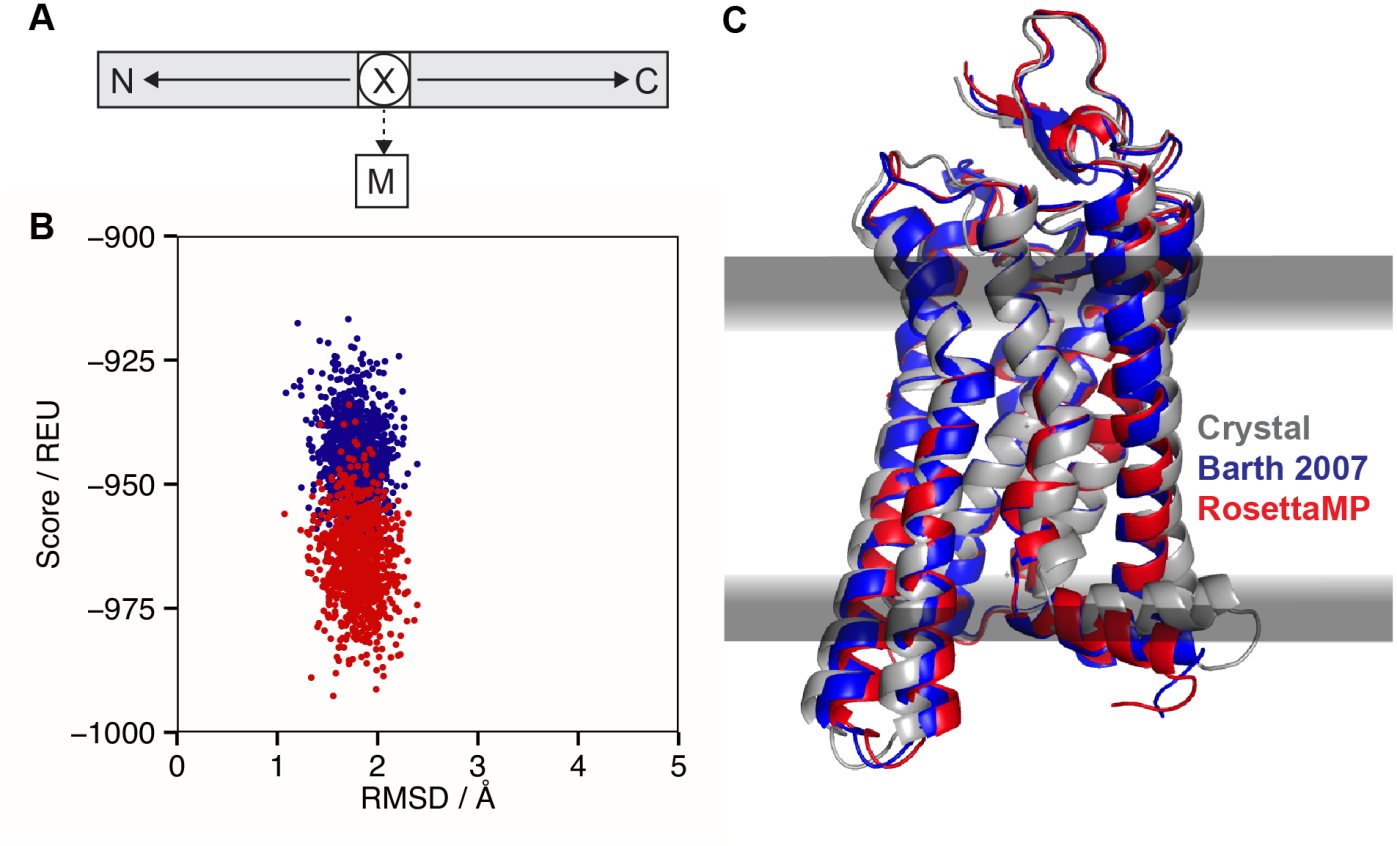
MPrelax for high-resolution refinement of a membrane protein. (A) FoldTree representation for the MPrelax protocol with the residue closest to the center-of-mass of the protein being at the root of the FoldTree (circled X). The membrane residue (M) is attached via a flexible jump edge (dashed arrow). Protein chains are shown as gray boxes with N- and C- termini marked and peptide edges shown as solid arrows. (B) Rosetta total score vs. backbone RMSD to the crystal structure for 1000 models of meta-rhodopsin. Models in blue are created with the original membrane relax protocol of RosettaMembrane; models in red are created with MPrelax. (C) Crystal structure of meta-rhodopsin in gray (PDB 3pxo) superimposed with the lowest scoring models from both the original RosettaMembrane protocol (blue) and the MPrelax protocol (red).

We combined RosettaMP with Rosetta’s high-resolution refinement protocol FastRelax [58,59], the high-resolution membrane score function [23,43], and a minimization-based technique for optimizing the protein embedding to create MPrelax (protocol capture in S3 File). The membrane residue is initially placed at the center-of-mass of the transmembrane spans and is allowed to move during the simulation by placing the FoldTree root in the protein (Fig 6A). The protein is then refined using FastRelax, performing eight iterations of rotamer trials and minimization of the backbone and side chains. During these iterations, the position and orientation of the membrane is optimized by gradient-based minimization.

We tested MPrelax on four test cases (Fig F in S1 File). As a representative example, Fig 6B and 6C show results for meta-rhodopsin II (PDB 3pxo) in the apo form [60] (calculations with retinal present are shown in Fig G in S1 File). For comparison, we also refined the structures with the original RosettaMembrane relax application [23]. The relaxed structures from both protocols are similar to each other and to the crystal coordinates (Fig 6C), and the new MPrelax protocol samples conformations at lower energies than the original RosettaMembrane relax (Fig 6B). The RosettaMP models have slightly different positions and orientations (embedding) in the membrane, since this method allows optimization of membrane embedding through minimization across the jump between the protein and the membrane residue. When RosettaMP outperforms RosettaMembrane, favorable hydrogen bonds result in lower scores (Table G in S1 File). Interestingly, the lower hydrogen bonding scores do not arise from an increase in the number of hydrogen bonds, but from better positioning within the membrane since the hydrogen bonding energy is dependent upon membrane embedding. In the original RosettaMembrane relax application, the membrane embedding is computed at the beginning of the protocol and then kept constant. For MPrelax, the minimization-based routine uses the membrane score function to optimize the position and orientation of the membrane based on the evolving protein conformation.

Similar to the meta-rhodopsin case, the new relax protocol also leads to lower scores in methyltransferase (PDB 4a2n) and histidine kinase receptor QseC (PDB 2kse) as shown in Supplementary Fig F and Tables H and I in S1 File. One more case (disulfide bond protein B, PDB 2leg, Table J in S1 File) shows higher scores with MPrelax than with the original relax protocol. Since this protein does not fully span the hydrophobic thickness of the membrane, this data suggests the minimization routine used by RosettaMP requires adjustment to sample a larger conformational space for embedding. For all cases, the range of sampled structures typically covers 1–6 Å RMSD, but often with a lack of structures close to native (within 1–2 Å). This may indicate that Rosetta scores the near native structures poorly, perhaps due to clashes in these structures, errors in the score function, or artifacts of the artificial lipid environment in the experiments that preclude these structures from being the low-energy conformation in an ideal, implicit, slab-like membrane.

#### Protein-protein docking in the membrane bilayer (MPdock)

**Fig 7:**
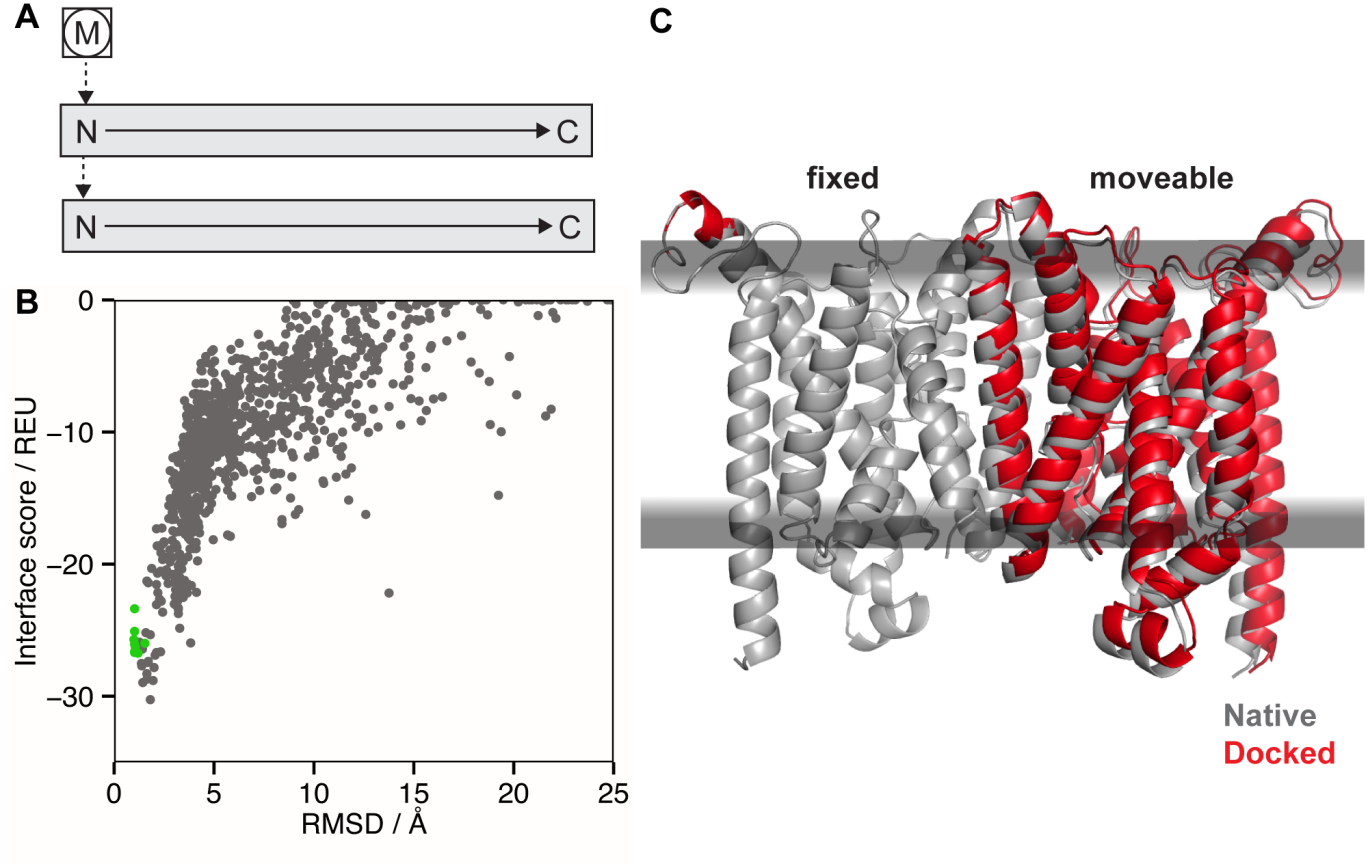
Protein-protein docking in the membrane bilayer using MPdock. (A) FoldTree representation used in MPdock with the membrane residue (M) being fixed at the root (circled) of the FoldTree and the protein chains as docking partners attached via jump edges. (B) Interface score vs. backbone RMSD to the native structure for 1000 models of the vitamin B12 importer BtuCD. The RMSD is the ‘ligand’ RMSD, which is computed only over the moving partner after superimposing the fixed partner and membrane. The green dots represent ten models created by minimizing the crystal structure. The interface score of the crystal structure (180.5 REU) is outside of the plotting range due to clashes. (C) Native structure of the vitamin B12 importer (gray, PDB 2qi9) superimposed with the model having the lowest interface score (red).

Structure determination of protein-protein complexes in the membrane bilayer is extraordinarily difficult due to the requirement for the membrane mimetic to maintain stability of the complex and because many complexes exceed the molecular weight limit for NMR spectroscopy. We combined RosettaMP with the RosettaDock algorithm [61,62] to predict structures of protein-protein complexes in the membrane bilayer (protocol capture in S4 File). The protocol consists of two steps: (1) a prepacking step to create a starting structure, and (2) protein-protein docking in the membrane bilayer. In the pre-packing step, the two partners are first separated by a large distance (keeping their membrane embedding constant), the side chains are repacked using rotamer trials, and the partners are moved back together. Next, the docking step samples random interface conformations using a score function that is created by combining the standard docking score functions with the membrane score terms (both in the low-resolution and all-atom stages, see Tables C-D in S1 File). The membrane bilayer is kept fixed during this simulation (Fig 7A).

MPdock was tested on five protein-protein complexes in the membrane. We created 1000 models for each complex and examined the structures, interface scores, fraction of native residue contacts, and RMSD values to the crystal structures (Fig H in S1 File). As an example, Fig 7 shows data for vitamin B12 importer BtuCD (PDB ID: 2qi9, docking partners are chains A and B), a dimer with 10 transmembrane helices per subunit. The score vs. RMSD plot in Fig 7B shows that the score function is able to distinguish near-native from non-native conformations; the model with the lowest interface score has an RMSD of 1.8 Å. Across our test set, the score function is able to distinguish native-like from non-native conformations in about half of the targets (Fig H in S1 File). In these cases, it recovers greater than 50% of native contacts meeting the CAPRI criteria for high quality predictions [63]. Scoring failures are seen for glycophorin A and for the SecYG complex, where the refined crystal structures have higher scores than the models with the lowest interface score. The native conformation for the methionine transporter has higher scores than the lowest scoring models, which have larger interfaces. These models suggest either valid alternate conformations of the transporter or imbalances in the score function. While these preliminary data are encouraging, it is important to note that these proof-of-concept calculations were restricted to a local vicinity of the interface in the crystal structure and that crystal backbone coordinates were used. Improvements to both the sampling routine as well as the score function are needed for docking of unbound complexes or homology models, or to carry out blind, global docking in the membrane environment while simultaneously moving both partners in the membrane.

#### Assembly of symmetric membrane protein complexes (MPsymdock)

**Fig 8:**
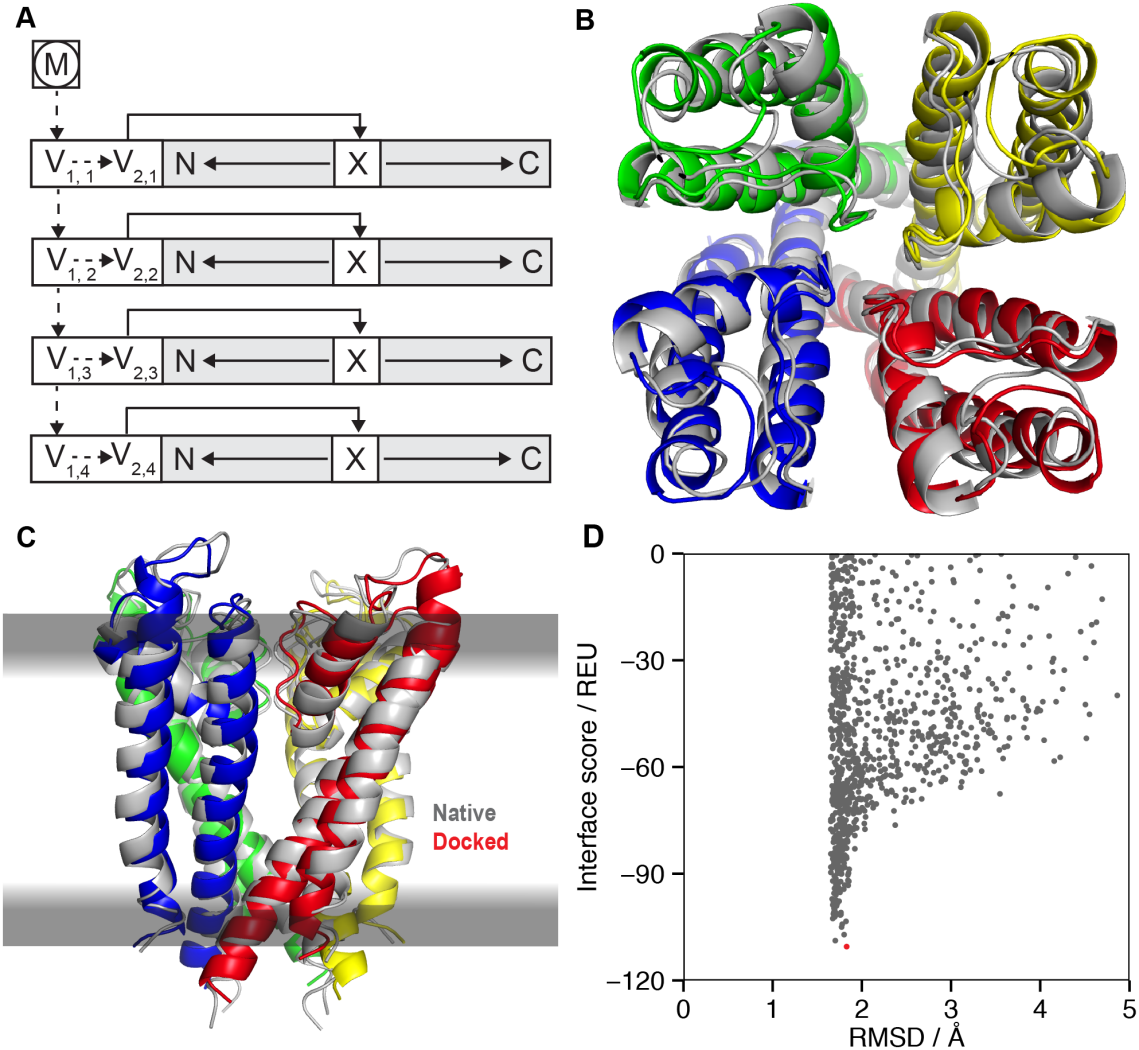
Assembly of symmetric protein complexes in the membrane using MPsymdock. (A) FoldTree representation of the homo-tetrameric KcsA potassium channel with the membrane residue (M) at the root (circled). The virtual residues V_1,i_ and V_2,i_ required for the symmetry machinery are described in the text. (B) Native structure in gray (PDB 1bl8) superimposed with the model from MPsymdock with the lowest interface score. The view is from the extracellular side of the membrane. (C) Membrane plane view of (B). (D) Interface score vs. backbone RMSD to the native structure for 1000 models of the KcsA potassium channel. The lowest scoring model, shown in (B) and (C), is indicated in red.

Many membrane proteins assemble into symmetric complexes in the membrane environment. We developed an application for symmetric assembly of complexes in the membrane bilayer; we achieved this by combining Rosetta’s symmetric docking protocol [64] with RosettaMP (Fig 8 with protocol capture in S5 File). The FoldTree maintains internal symmetry of the complex and its position in the membrane bilayer, with the membrane residue being at its root, hence keeping it fixed (Fig 8A). The subunits are arranged in C_*n*_ symmetry around the membrane normal axis (defined consistently in this protocol as the *z*-axis), where *n* is the number of subunits in the complex. To account for symmetry in the protein, the FoldTree uses two additional virtual residues per subunit, V_1,i_ and V_2,i_, where *i* is the number of the subunit and 1 ≤ *i* ≤ *n*. The jump from *V*_1,i_ to *V*_1,i+1_ describes the rotation and translation required to transform the *i* ^th^ subunit to the (*i*+1)^th^ subunit based on the C_*n*_ symmetry, and the jump from *V*_*1*,*i*_ to *V*_*2*,*i*_ describes the rotation and translation between the *V*_*1*,*i*_ and the protein subunit root residue. This setup allows the protocol to respect both symmetry and the membrane environment while allowing efficient sampling moves, side chain packing, and scoring.

MPsymdock was tested on four examples (Fig I in S1 File) with one example shown in Fig 8: the crystal structure of the homo-tetrameric KcsA potassium channel (PDB 1bl8). Because the membrane protein docking score function had a low tolerance for initial clashes in the structure, each complex was first refined using the MPrelax protocol described above. Ten models were generated, and one asymmetric unit of the lowest scoring model was used as input to the symmetric docking protocol. From the asymmetric unit, the full complex was reassembled by the symmetric docking routine and the interface score was computed as the cumulative score of all symmetric interfaces [65]. The interface score vs. RMSD plot (Fig 8D) indicates that the high-resolution membrane score function, adapted for symmetric docking, is able to distinguish near-native from non-native conformations; the model with the lowest interface score has an RMSD of 1.8 Å to the native crystal structure. This is an encouraging result, especially since for this target the interface in the selectivity filter contains a number of proximal carbonyl oxygen atoms. The large interface size between the subunits may compensate for adverse effects in the selectivity filter. This example suggests the requirement for a more sophisticated membrane model accounting for channels in the membrane. Glycophorin A is similarly successful (Fig I in S1 File). In both cases, the RMSD is elevated by the asymmetry in tail regions of each subunit in the native reference structure; calculations without the tails may be more physically realistic and produce lower RMSDs.

While the low-RMSD docked complexes of KcsA and glycophorin A are encouraging, the remaining two examples were unable to form native-like complexes with well-packed interfaces; instead the subunits separated during the search to relieve clashes (Fig I in S1 File). To obtain high-quality models, several improvements are necessary. A new symmetric refinement protocol could generate symmetric starting structures while removing clashes [66]. Another approach to remove clashes would be through backbone flexibility in the protocol itself; this approach will require a gradual transition to introduce repulsive van der Waals energies to avoid the high gradients that currently cause the subunits to separate.

## Discussion

Here, we describe the use of a software suite for biomolecular modeling, docking, and design to enable rapid development of new applications targeted at membrane proteins, a class for which structure determination efforts are notoriously difficult. We used the scientific concepts in the RosettaMembrane structure prediction and refinement applications and created a modular framework within Rosetta3’s object-oriented architecture [32]. The four proof-of-concept applications demonstrate flexibility, generality, and simplicity of RosettaMP. The new framework enables combination of the membrane environment with a variety of Rosetta features: with the fold tree [36], jumps in membrane proteins can be used to model multiple protein chains, flexible loops, and ligands; and with symmetry [64], symmetric protein structure prediction, refinement and design will be feasible. RosettaMP will serve as the starting point for future protocol development, and each new application can be extensively tested and benchmarked. Our preliminary results show that RosettaMP has the potential to answer long-standing questions involving membrane proteins and lays the groundwork for the challenges that still remain.

RosettaMP complements many existing tools for membrane protein modeling. MPrelax can be used to refine proteins inserted into the membrane using tools such as iMembrane [67]. MPrelax can also be used in combination with a contact prediction method to predict structures with low sequence similarities to their template (similar to I-TASSER [68,69]). MPddG can directly be used for alanine scanning and extended for membrane protein- and interface-design. Homology models from MEDELLER [70] and Rosetta [16] can be used as input to MPdock and MPsymdock for modeling of large membrane protein complexes. In principle, the MPsymdock protocol can also be used to distinguish biological interfaces from non-native crystal contacts (similar to COMP [71] or PISA for soluble proteins [72]). The variety of these potential applications shows that RosettaMP forms an important basis for new protocol development.

The key components of any structure prediction or design algorithm are sampling and scoring. Conformational sampling routines are improved via RosettaMP through the connection of the membrane bilayer to the modeled biomolecule. This representation allows flexibility in choosing which object should be fixed vs. movable (protein or membrane) by representing the membrane as a ‘residue’ and using a jump in the fold tree. For example, a fixed bilayer enables sampling of membrane-embedded docking conformations in the MPdock and MPsymdock protocols, whereas a movable membrane decreases the computational cost of the MPrelax protocol. The latter allows optimizing the membrane position and orientation using minimization algorithms, resulting in lower scores for three of four cases. Moreover, the flexible linkage now permits constraining spans, chains, or proteins to the membrane in various depths and orientations, features that could not be modeled previously. The framework also simplifies implementation of enhanced sampling protocols through specialized movers that favor meaningful protein conformations in the membrane, for instance for *ab initio* prediction of α-helical and β-barrel membrane proteins using particular fragment types or favoring appropriate orientations and pairings of the secondary structure elements.

For scoring, the new framework allows us to test Rosetta’s membrane score functions in new contexts. The four applications collectively demonstrate that the existing low- and high-resolution membrane protein score functions are generally effective, yet require further optimization. The preliminary MPddG application is able to identify favorable vs. disruptive mutations in the two tested cases and even produces a reasonable correlation for predicted vs. experimental ΔΔ*G* values for an intra-membrane residue in OmpLA. Refinement, which is minimally modified from its original, tested implementation, captures the same minima. The naïve score functions for docking and symmetric docking exhibit minima for native-like interfaces in about half of the asymmetric and symmetric cases. These results are encouraging, especially since we have not made any changes to the RosettaMembrane score functions originally developed for folding and refinement.

For future work, several improvements to the score function seem possible. Since the number of determined membrane protein structures has increased substantially in recent years, the low-resolution, knowledge-based score functions can be updated to reduce statistical errors. Further, existing score functions were solely derived from α-helical membrane proteins, and data from β-barrels could be used to create a distinct score function that could be tested with large-scale folding and refinement of these proteins. For instance, a recently derived hydrophobic potential for outer membrane β-barrels has been found to be condensed compared to that for α-helical membrane proteins, since bacterial outer membranes have a smaller membrane thickness [55,57]. An updated formulation of Rosetta’s distance-dependent dielectric electrostatic score [73] is needed to accommodate the low dielectric constant in the membrane. It is also now feasible in principle to sample protonated and deprotonated forms of ionizable residues with Rosetta pH [74,75]; however parameterization is needed to account for the insertion of charged species in the membrane. An advantage of RosettaMP is that new score functions can now be more easily derived than previously by using the score function machinery in Rosetta3.

The current membrane model is a flat bilayer model of fixed thickness, i.e. a slab model. RosettaMP could be a stepping-stone for tackling complex biological questions with more sophisticated membrane models. Effects needed to create the next generation of this model include intrinsic curvature, charge asymmetry [76–78], and variable thickness [79], attributed to the diverse repertoire of lipids that constitute the membrane environment [80]. Membrane thickness might play a role in the accurate estimation of ΔΔ*G* values for interfacial aromatic residues [51] and in reproducing snorkeling of arginine and lysine to the membrane interface [54,81]. More sophisticated membrane models will be required for proteins that form pores or toroidal pores [82]. Another challenge is the modeling of membrane-anchored proteins or peptides [83] especially for small and/or unstructured peptides or half-helices inserting into the bilayer that are not identified as such by sequence-based prediction methods.

RosettaMP will also enable the development of design protocols, an important yet challenging task with potential impact in synthetic biology and gene therapy. The latest membrane protein design efforts focused on helix-helix interfaces [20], protein chimeras [84], and used protein display [85] and combinatorial libraries [86] to identify promising designs. These efforts require manual processes and use full-atom energy functions derived mostly from MD force fields. With robust score functions and sampling routines, methods developed with RosettaMP will add to these emerging tools and complement MD simulation packages, enabling investigation of membrane protein structure, dynamics, and function from low- to high-resolution representations.

In summary, we anticipate new progress by combining the power of existing Rosetta applications with RosettaMP. By making membrane protein modeling and design accessible to the broad scientific community, we hope to drive understanding of membrane protein structure, function and ultimately enable drug design for this essential class of proteins.

## Supporting Information

S1 File Object-oriented design, detailed methods, and results. This file contains a discussion of the object-oriented design of the RosettaMP framework. Both the low-resolution and high-resolution score functions are described in detail. Finally, we discuss detailed methods and results for development and testing of each RosettaMP application.
(PDF)

S2 File Protocol capture for MPddG. This protocol capture contains the steps, input files and example output files necessary to run the MPddG protocol described in this manuscript. While both mutations in OmpLA and OmpA are discussed, we only describe the protocol for computing ΔΔ*G*s for OmpLA for simplification. The supplementary files are included with the Rosetta3 software suite under the directory Rosetta/demos/protocol_capture/MPddG.
(GZ) Click here for additional data file.

S3 File Protocol capture for MPrelax. This protocol capture contains the steps, input files, and example output files necessary to run the MPrelax protocol described in this manuscript. For simplification, we only describe refinement of meta-rhodopsin II in these files. The supplementary files are included with the Rosetta3 software suite under the directory Rosetta/demos/protocol_capture/MPrelax.
(GZ) Click here for additional data file.

S4 File Protocol capture for MPdock. This protocol capture contains the steps, input files, and example output files necessary to run the MPdock protocol described in this manuscript. For simplification, we only describe refinement of Glycophorin A in these files. The supplementary files are included with the Rosetta3 software suite under the directory Rosetta/demos/protocol_capture/MPdock.
(GZ) Click here for additional data file.

S5 File Protocol capture for MPsymdock. This protocol capture contains the steps, input files, and example output files necessary to run the MPdock protocol described in this manuscript. For simplification, we only describe docking of Glycophorin A in these files. The supplementary files are included with the Rosetta3 software suite under the directory Rosetta/demos/protocol_capture/MPdock.
(GZ) Click here for additional data file.

S6 File Protocol capture for MPPyMOLViewer. This protocol capture contains the steps, input files, and example output files necessary to run the MPPyMOLViewer protocol described in this manuscript. For simplification, we only describe visualization of bacteriorhodopsin these files. The supplementary files are included with the Rosetta3 software suite under the directory Rosetta/demos/protocol_capture/MPPyMOLViewer.
(GZ) Click here for additional data file.

S7 File Protocol capture for MPSpanFromPDB. This protocol capture contains the steps, input files, and example output files necessary to run the MPPyMOLViewer protocol described in this manuscript. For simplification, we only describe visualization of bacteriorhodopsin. The supplementary files are included with the Rosetta3 software suite under the directory Rosetta/demos/protocol_capture/MPSpanFromPDB.
(GZ) Click here for additional data file.

S1 Movie Real-Time Visualization of Membrane Simulation in PyMOL. Visualization of a symmetric docking simulation of the protein complex potassium channel KcsA (PDB ID 1bl8). For every conformational change in the simulation, Rosetta sends an updated structure to PyMOL via the PyMOL Mover. The new structure is displayed in real-time in PyMOL. Asymmetric subunits colored independently. The complex is first assembled from a single asymmetric subunit, docked in low-resolution, and then refined in high-resolution.
(MP4)
