# Supplementary material for: An Integrated Framework Advancing Membrane Protein Modeling and Design
Source: PLoS Comput Biol. 2015 Sep 1;11(9):e1004398. doi: 10.1371/journal.pcbi.1004398 (PMC4556676; doi:10.1371/journal.pcbi.1004398)
Supplement: S1 File — This file contains a discussion of the object-oriented design of the RosettaMP framework. Both the low-resolution and high-resolution score functions are described in detail. Finally, we discuss detailed methods and results for development and testing of each RosettaMP application. (PDF) [file pcbi.1004398.s001.pdf]

Supplementary Material for

"An integrated framework advancing membrane protein modeling and design"

RF Alford, J Koehler Leman, BD Weitzner, AM Duran, DC Tilley, A Elazar, JJ Gray

Table of Contents

RosettaMP design relies on object-oriented programming concepts 2

Membrane score functions 5

Detailed Methods and Results 10
- **Model preparation 10**
- **MPddG: Prediction of free energy changes (ΔΔG) in the membrane 10**
  - Contributing Rosetta energies to predicted ΔΔG values 10
  - Detailed methods and command lines 15
- **MPrelax: High-resolution membrane protein refinement 17**
  - Additional test cases 17
  - Origin of differences between RosettaMembrane (Barth, 2007) and RosettaMP refinement 18
  - Detailed methods and command lines 21
  - Refinement of Metarhodopsin II (PDB 3pxo) with Retinal 24
  - Detailed methods and command lines 25
- **MPdock: Membrane protein-protein docking 26**
  - Additional test cases 26
  - Detailed methods and command lines 28
- **Application MPsymdock: Assembly of symmetric membrane protein complexes 29**
  - Additional test cases 29
  - Detailed methods and command lines 31
- **Membrane visualization 32**
  - Detailed command lines 32
- **Application MPspanfrompdb: Calculate transmembrane spans from structure 33**
  - Detailed methods and command lines 33

References 33

RosettaMP design relies on object-oriented programming concepts

Today, robust algorithms are not the only requirement for high-level scientific software. Software designs must incorporate features for multiple tasks within a single platform. Several tools have already benefited from this integrative approach, including the Integrative Modeling Platform [1], Chimera visualization toolset [2], Biopython package [3], Phenix [4] and the Rosetta software suite [5]. Each of these platforms houses an architecture that permits the user to interchange components, thereby creating a new set of complex tools for biomolecular modeling, design, and visualization. This flexible and expansive nature of software enables these computational tools to answer a broader range of scientific questions than ever before.

Object-oriented design principles in RosettaMP

To create an integrated environment for membrane protein modeling, we applied object-oriented programming concepts [6] to the design of RosettaMP. Specifically, we relied on the concepts below to extend the existing architecture Rosetta3 [7]:

- **Cohesion:** The methods and data in a single object should represent a single concept in detail
- **Extension (Inheritance):** Create an object that is a subtype of a parent object. This new object inherits parent methods and data, as well as defining its own features
- **Containment (Encapsulation)**: Store a new object (or multiple of that object) as data in an existing object
- **Template:** Define an abstract class that defines data to be filled (the template). During creation, fill the data depending on what is being represented.
- **Composite**: Represent that objects stored in the parent object have a part-to-whole relationship
- **Mediation**: Track another objects for updates

Each principle described aided the extension of the scoring and sampling infrastructure in Rosetta3, as well as creating new objects for representing biomolecules in the membrane. Principles applied to the design of each object (or set of objects) in RosettaMP are listed in Table A.

Table A: Object-oriented programming concepts in RosettaMP. Object-oriented programming concepts applied to the design and placement of objects in the RosettaMP framework.

Object	Purpose	Design principle
`MembraneInfo`	Store and track information required for representing the membrane bilayer during a simulation	Encapsulation, Mediation
`Span`	Start and end residue numbers of an individual transmembrane span	Composite (part)
`SpanningTopology`	Group of transmembrane `Span` objects describing topology of the whole `Pose`	Composite (whole)
`Membrane Residue`	Fill coordinates and properties in a `Residue` object to define the current position of the membrane bilayer	Template, Mediated by MembraneInfo
`Membrane Jump`	Define the current orientation of the membrane bilayer with respect to the biomolecule(s). Allow the bilayer to be fixed or moveable	Template, Mediated by MembraneInfo
`Membrane EnergyMethods`	Individual membrane-specific scoring terms	Inheritance
`Membrane Movers`	Sample conformations with respect to the position and orientation of the membrane bilayer	Inheritance

The object-oriented architecture of the `ScoreFunction`, `EnergyMethod` hierarchy and `Movers` have been previously described in [7]. Because the membrane representation objects are completely new, we describe the architecture of these objects in Fig. A.

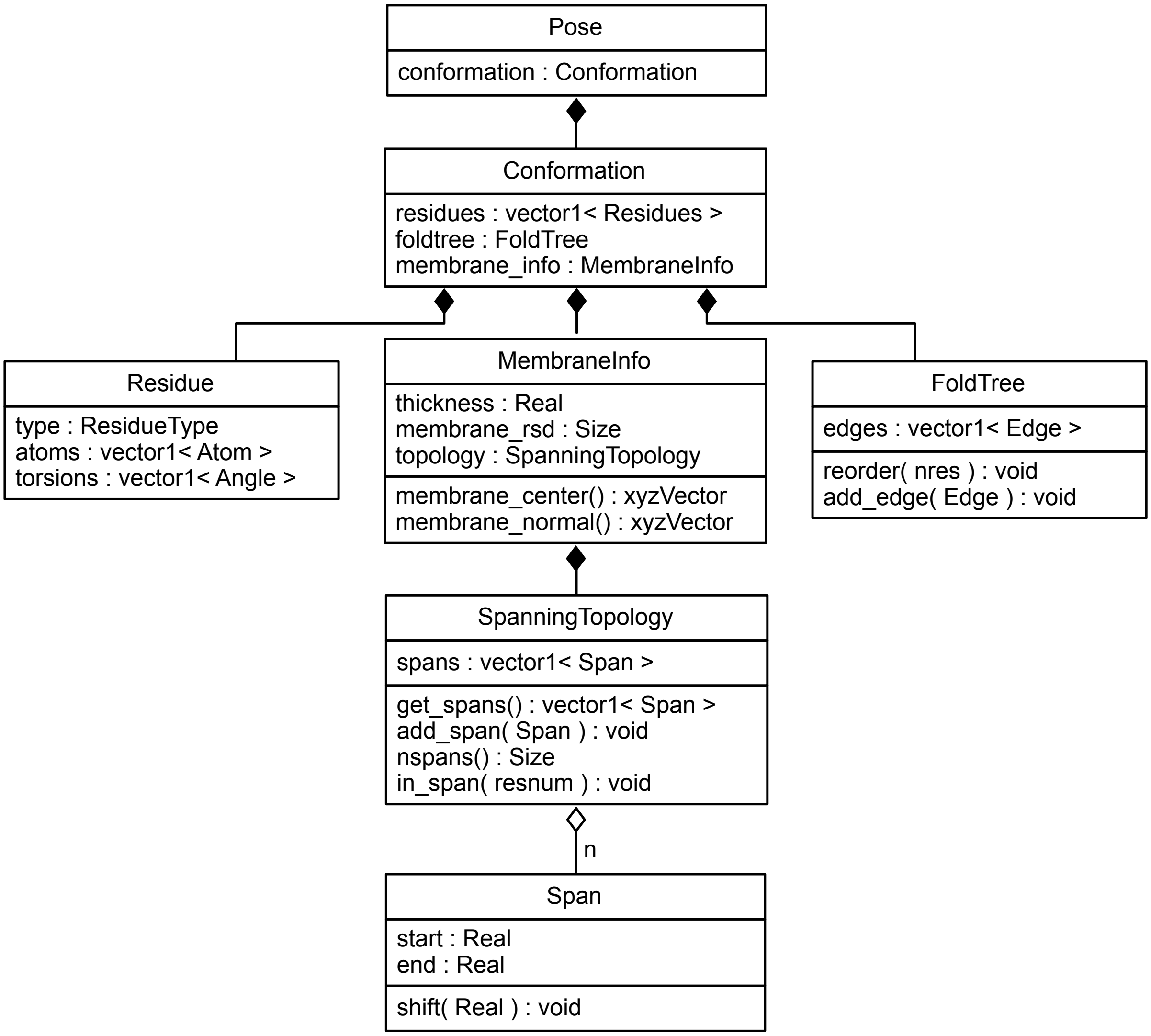

Fig. A: Detailed architecture of the membrane representation used in the framework
The object-oriented architecture of the membrane representation is described with Unified Modeling Language (UML). Each box describes an individual object. The top field specifies the object name, the middle field specifies data stored by the object, and the bottom field describes methods in that object. Filled diamond arrows denote one object is contained in the other. Open diamond arrows denote *n* objects are contained in the parent object.

Expanding Rosetta's residue type library to include a Membrane Residue

The Rosetta database includes an extensive library of residues: the individual chemical building blocks that make up a biomolecule [7,8]. The wide range of chemical building blocks, ranging from canonical amino acids to small molecules, is described by encapsulating a `ResidueType` template object in the `Residue` object. A `ResidueType` defines properties specific to that residue, and can be added to Rosetta by including a new Residue parameter file to the database. The parameter file for the membrane residue is described in Fig. B.

A

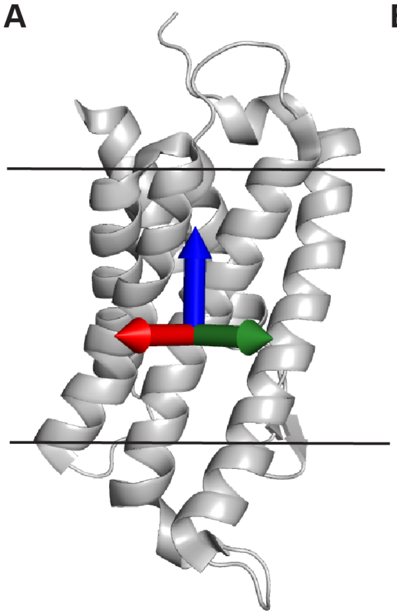

B

```
# Rosetta Residue Topology File
NAME MEM
IO_STRING MEM Z
TYPE LIGAND
AA UNK

PROPERTIES MEMBRANE
ATOM CNTR MPct H     0.0
ATOM NORM MPnm H     0.0
ATOM THKN MPtk H     0.0
BOND CNTR NORM
BOND CNTR THKN

ICOOR_INTERNAL CNTR   0.000   0.000  0.000
ICOOR_INTERNAL THKN  90.000 180.000 15.000
ICOOR_INTERNAL NORM 270.000  90.000 15.000

NBR_ATOM CNTR
NBR_RADIUS -100000.0
FIRST_SIDECHAIN_ATOM ALL
```

Fig. B: Rosetta residue parameter file describing membrane residue parameters
To represent the geometry of the membrane bilayer, we added a new `ResidueType` to the Rosetta chemical database. This small conformational building block does not chemically interact with other residues and describes the spatial orientation of the membrane. (A) Coordinate frame describing the membrane geometry contained in the membrane residue (B) Rosetta residue parameter file describing initial parameters for the membrane residue.

The residue parameter file for the centroid MEM residue can be found in: `Rosetta/database/chemical/residue_type_sets/centroid/residue_types/membrane/MEM.params`

The residue parameter file for the full atom MEM residue can be found in: `Rosetta/database/chemical/residue_type_sets/fa_standard/residue_types/membrane/MEM.params`

Membrane score functions

A low- and high-resolution score function were introduced as part of the original RosettaMembrane implementation [9–11]. To make these score functions compatible with RosettaMP, we created a new set of `EnergyMethod` objects for each score term. Each score term preserves the scientific integrity of the original energy function, while also relying on the membrane representation in RosettaMP for scoring.

The low-resolution and high-resolution terms use different representations of the membrane during scoring. At the low-resolution stage, the membrane comprises of five discrete layers to describe the hydrophobic core, interface, and solvent regions [9]. In contrast, the full-atom energy uses the implicit membrane model (IMM1) described by Lazaridis [12] to describe a continuous dielectric gradient from the hydrophobic core to solvent region. This membrane model includes a hydrophobic layer, transition region, and soluble region.

The functional form of each term supported by RosettaMP is shown Table B.

Table B: Functional forms of Rosetta membrane energy terms

Term	Functional Form	Parameters	Ref
`mp_env`	$E_{env} = \sum_i -\ln\left(\frac{P(aa_i \mid L,B)}{P(aa_i)}\right)$	i = residue index<br>aa= amino acid type<br>L = layer in the membrane<br>B = burial state in the protein	[9]
`mp_pair`	$E_{pair} = \sum_i \sum_{j>i} -\ln\left(\frac{P\left(\mathrm{aa}_i \mathrm{aa}_j \middle\| d_{ij}, L\right)}{P\left(\mathrm{aa}_i \middle\| d_{ij}, L\right) P\left(\mathrm{aa}_j \middle\| d_{ij}, L\right)}\right)$	i,j = residue indices<br>aa= amino acid type<br>d = distance between residues<br>L = layer in the membrane	[9]
`mp_cbeta`	$E_{density} = \sum_i \sum_{sh} -\ln\left(\frac{P_{compact}(nb_{i,sh})}{P_{random}(nb_{i,sh})}\right)$	i = residue (centroid) index<br>sh = shell radius, 6 Å or 12 Å<br>nb = number of neighboring residues within a shell<br>P_{compact} = probability in compact structure assembled from fragments<br>P_{random} = probability in random structure from fragments	[9,13]
`mp_nonhelix`	$E_{nonhelix} = 10 * \sum_i \begin{cases} 1 & if\ (-12 < z < 12) \text{ and } (ss \neq \text{H}) \\ 0 & else \end{cases}$	i = residue index<br>z = residue depth in membrane<br>ss = secondary structure type (Helix (H), Strand (S) or Coil (C))	[9]
`mp_termini`	$E_{termini} = 50 * \sum_i \begin{cases} 1 & if\ (-12 < z < 12) \\ 0 & else \end{cases}$	i = residue index<br>z = residue depth in membrane	[9]
`mp_tmproj`	$E_{tmproj} = 50 * \sum_h \begin{cases} 2 & if\ (l < 15)\ and\ (r < 1.0\ or\ r > 1.5) \\ 1 & if\ (l < 15)\ or\ (r < 1.0\ or\ r > 1.5) \\ 0 & else \end{cases}$	h = transmembrane (TM) helix index<br>l_h = TM helix length in Ångströms<br>r_h = l_h / number of residues in TM helix	[9]

Term	Functional Form	Parameters	Ref
`fa_mpenv`	$E_{env} = \sum_{i}^{N_{atom}} \Delta G_i^{ref}(z')$<br><br>$\Delta G_i^{ref}(z') = (1 - f(z')) \cdot (\Delta G_i^{ref,chex} - \Delta G_i^{ref,water})$	i = atom index<br>$z' = \lvert z \rvert/(T\ /\ 2)$, where T is the membrane thickness<br>ΔG_i^{ref}= free energy of an isolated atom in the membrane<br>N_{atom} = number of atoms in the molecule	[10,12]
`fa_mpsolv`	$E_{solv} = \sum_{i}^{N_{atom}} \sum_{j>i}^{N_{atoms}} \left\{ \frac{2\Delta G_i^{free}}{4\pi\sqrt{\pi}\lambda_i {r_{ij}}^2} \exp\left(-{d_{ij}}^2\right) V_j + \frac{2\Delta G_j^{free}}{4\pi\sqrt{\pi}\lambda_j {r_{ji}}^2} \exp\left(-{d_{ji}}^2\right) V_i \right\}$<br><br>$\Delta G_i^{free}(z') = f(z') \cdot \Delta G_i^{free,water} + (1 - f(z'))\Delta G_i^{free,chex}$	i,j = atom indices<br>d = distance between atoms<br>r = sum of van der Waals radii<br>V = atomic volume<br>λ = correlation length<br>ΔG^{free}= solvation free energy of the free (isolated) atom<br>N_{atom} = number of atoms in residue i<br>N_{atoms} = number of atoms in each residue j within 12 Å of atom i	[10,12]
`fa_mpenv_ smooth`	$E_{env} = \sum_{i} -\ln\left(\frac{P(aa_i \mid L, B)}{P(aa_i)}\right)$	centroid term with the same functional form as the centroid term `mp_env`; this term is derived from a full-atom representation and can only be used in full-atom mode	[11]
`hbond`	$E_{hbond} = W_{hbond} * W_{burial}^{hbond}(z', nb) * E_{geo}^{hbond}(d, \Psi, \Theta)$<br><br>$W_{burial}^{hbond}(z', nb) = f(z') \cdot W_{burial,water}^{hbond}(nb) + \left(1 - f(z')\right) \cdot W_{burial,membrane}^{hbond}(nb)$	nb = number of neighboring residues within a shell<br>z' = depth of atom pairs in the membrane<br>W_{hbond} = global weight optimized by sequence recovery and decoy features<br>$E_{geo}^{hbond}(d, \Psi, \Theta)$ = orientation dependent hydrogen bonding energy<br>$W_{burial}^{hbond}(z, nb)$ = membrane dependent hydrogen bonding weight	[10,14]

$f(z')$ describes the transition between the phases and is defined as $f(z') = z'^n/(1 + z'^n)$ with n controlling the steepness of the transition.

For scoring in the membrane environment, each application relies on a different combination of terms and weights. The MPrelax and MPddG protocols use the standard set of weights for the high-resolution membrane energy function. The MPddG with pH scoring, MPdock and MPsymdock application uses a special weights set. All weights used in this manuscript are described in Tables C and D.

The standard weights set for the **low-resolution** membrane score function can be found in:
`Rosetta/main/database/scoring/weights/mpframework_cen_2006.wts`

The **original high-resolution** membrane score function can be found in:
`Rosetta/main/database/scoring/weights/mpframework_fa_2007.wts`

This is superseded by the standard weight set for the **high-resolution** membrane score function, also used for **MP_Relax** and **MP_ddG**, which can be found in:
`Rosetta/main/database/scoring/weights/mpframework_smooth_fa_2012.wts`

Weights used for the **MP_Dock** protocol can be found in:
`Rosetta/main/database/scoring/weights/mpframework_docking_cen_2015.wts`
`Rosetta/main/database/scoring/weights/mpframework_docking_fa_2015.wts`

Weights used for the **MP_SymDock** protocol can be found in:
`Rosetta/main/database/scoring/weights/mpframework_symdocking_cen_2015.wts`
`Rosetta/main/database/scoring/weights/mpframework_symdocking_fa_2015.wts`

Table C: Low-Resolution (centroid) score function weights used in RosettaMP applications.

Term	Yarov-Yarovoy Low Res 2006	MPdock Low Res 2015	MPsymdock Low Res 2015
mp_pair	1.000	1.000	1.000
mp_env	2.019	2.019	2.019
mp_cbeta	2.500	2.500	2.500
mp_nonhelix	2.019	-	-
mp_termini	2.019	-	-
mp_tmproj	2.019	-	-
rsigma	1.000	1.000	1.000
sheet	1.000	1.000	1.000
ss_pair	1.000	1.000	1.000
hs_pair	1.000	1.000	1.000
rama	0.150	0.150	0.150
interchain_env	-	0.100	1.000
interchain_pair	-	0.100	1.000
interchain_contact	-	0.200	1.000
interchain_vdw	-	10.000	1.000

Table D: High-Resolution (all-atom) score function weights used in RosettaMP applications.

Term	Barth High Res 2007	High Res 2012	MPddG 2015	MPdock High Res 2015	MPsymdock High Res 2015
fa_atr	0.780	0.800	0.800	0.800	0.800
fa_rep	0.430	0.440	0.440	0.440	0.440
fa_intra_rep	0.004	0.004	0.004	0.004	0.004
fa_pair	0.480	0.490	0.490	0.490	0.490
fa_dun	0.560	0.560	0.560	0.010	0.560
fa_mpenv	0.480	0.300	0.300	0.300	0.300
fa_mpenv_smooth	-	0.500	0.500	0.500	0.500
fa_mpsolv	0.600	0.350	0.350	0.350	0.350
ref	1.000	1.000	1.000	1.000	1.000
hbond_lr_bb	1.160	1.170	1.170	1.170	1.170
hbond_sr_bb	1.160	1.170	1.170	1.170	1.170
hbond_bb_sc	1.160	2.340	2.340	2.340	2.340
hbond_sc	1.100	2.200	2.200	2.200	2.200
p_aa_pp	0.640	0.320	0.320	0.320	0.320
dslf_ss_dst	0.500	0.500	0.500	0.500	0.500
dslf_cs_ang	2.000	2.000	2.000	2.000	2.000
dslf_ss_dih	5.000	5.000	5.000	5.000	5.000
dslf_ca_dih	5.000	5.000	5.000	5.000	5.000
pro_close	1.000	1.000	1.000	1.000	1.000
rama	0.200	0.200	0.200	0.200	0.200
omega	0.500	0.500	0.500	0.500	0.500
fa_elec	-	-	0.875	0.026	0.026

Standard high-resolution weights were used for the MPrelax and MPddG applications

Detailed Methods and Results

Model preparation

For cases in each application, The structure each protein was downloaded from the Protein Databank of Transmembrane Proteins website (PDBTM: *http://pdbtm.enzim.hu/*) [15]. Protein structures from this database are transformed into a reference membrane coordinate frame – providing a consistent set of models with starting orientations in the bilayer. PDB files were cleaned and renumbered using the `clean_pdb.py` script in the `Rosetta/tools/protein_tools/scripts` directory. Spanfiles were generated using the `span_from_pdb` application described below.

MPddG: Prediction of free energy changes (ΔΔG) in the membrane

MPddG combines the RosettaMP framework with a fixed-backbone Δ*ΔG* prediction protocol, similar to the method described in Kellogg *et al*. [16]. For each Δ*ΔG* prediction, side-chain conformations for residues within 8 Å of the mutant position are sampled and the Δ*ΔG* is taken as the difference in Rosetta Energy Units (REU) between the mutant and native conformation.

Contributing Rosetta energies to predicted ΔΔG values

We investigated the contribution of each energy term to the overall Δ*ΔG* by taking the difference in individual scores between the mutant and native conformation. Data for mutations in OmpLA and OmpA are shown in Table E and F respectively. In both tables, scores are colored using a blue to red gradient, where blue indicates a favorable score ($\Delta\Delta G < 0$) and red indicates an unfavorable score ($\Delta\Delta G > 0$). In each row, the most saturated values are the minimum and maximum values. Energies with no contribution to the Δ*ΔG* were excluded from both tables. These are the disulfide bonding energies (dslf_ss_dih, dslf_ca_dih, dslf_cs_ang, and dslf_ss_dst), hydrogen bonding energies (hbond_sr_bb, hbond_lr_bb, hbond_bb_sc, and hbond_sc) and omega dihedral energy (omega). The role of structural features in predicted Δ*ΔG* values is also described in Fig. C-E.

Table E: Contribution of individual Rosetta scores to predicted $\Delta\Delta G$ values for mutations in OmpLA

mutation	total	fa_atr	fa_rep	fa_intra_rep	fa_elec	pro_close	fa_mpsolv	rama	fa_dun	p_aa_pp	ref	fa_mpenv	fa_mpenv_smooth
A210I	-0.882	-1.539	0.996	0.023	-0.087	0.000	0.292	-0.184	1.572	-0.399	0.080	-0.736	-0.900
A210L	-0.590	-1.588	0.414	0.028	-0.139	0.000	0.271	-0.082	2.543	-0.201	-0.260	-0.736	-0.840
A210M	-0.517	-0.863	0.578	0.003	-0.127	0.000	0.158	-0.218	1.375	-0.276	-0.500	-0.291	-0.356
A210V	-0.480	-1.293	0.744	0.018	0.020	0.000	0.248	-0.258	1.453	-0.489	0.130	-0.499	-0.554
A210N	3.373	-1.876	0.709	0.007	0.279	0.000	0.232	-0.035	2.166	-0.053	-1.050	1.835	1.159
A210Q	2.849	-3.084	1.257	0.009	-0.048	0.000	0.311	-0.131	3.322	-0.201	-1.130	1.597	0.947
A210S	0.302	-0.195	-0.032	0.002	-0.518	0.000	0.066	-0.125	0.223	-0.148	-0.530	1.161	0.398
A210T	1.272	-0.822	1.468	0.006	0.062	0.000	0.198	-0.135	0.120	-0.308	-0.430	0.900	0.213
A210F	0.704	-3.943	2.192	0.040	-0.044	0.000	0.413	-0.196	3.425	-0.239	0.470	-0.509	-0.905
A210W	0.903	-7.043	4.932	0.024	-0.146	0.000	0.736	-0.167	2.402	-0.161	0.750	0.308	-0.732
A210Y	1.485	-4.221	2.445	0.040	-0.062	0.000	0.703	-0.205	3.502	-0.337	0.350	-0.509	-0.222
A210C	1.409	-0.446	0.111	0.002	-0.202	0.000	0.110	-0.198	0.203	-0.230	1.540	0.355	0.164
A210G	1.680	0.820	-0.129	0.000	0.011	0.000	-0.122	0.405	0.000	0.359	-0.330	0.408	0.258
A210P	193.228	-1.885	52.357	0.001	0.082	138.896	0.562	1.164	1.461	1.042	-0.140	-0.831	0.519
A210R	2.568	-0.972	0.876	0.008	-0.052	0.000	0.163	-0.192	1.363	-0.259	-1.140	2.563	0.210
A210K	3.290	-0.891	1.111	0.004	-0.069	0.000	0.154	-0.147	0.932	-0.209	-0.810	2.251	0.964
A210D	5.029	-1.933	0.712	0.007	0.192	0.000	0.289	0.062	1.996	0.177	-0.830	2.631	1.725
A210E	4.859	-1.045	0.950	0.005	-0.359	0.000	0.191	-0.147	2.069	-0.126	-0.970	2.394	1.895
A210H	3.673	-3.560	0.292	0.020	0.287	0.000	0.413	-0.180	3.738	-0.207	0.400	2.005	0.358

Mutations are grouped by the following amino acid categories (from top to bottom): non-polar, polar, aromatic, special, positively charged and negatively charged. Contributions of individual terms are discussed in detail in the main text. Positive values (red) indicate alanine is more favorable and negative values (blue) indicate the guest side chain is more favorable.

The effects of the side chains for arginine/lysine, serine/threonine and the aromatic residues are shown in the figures below.

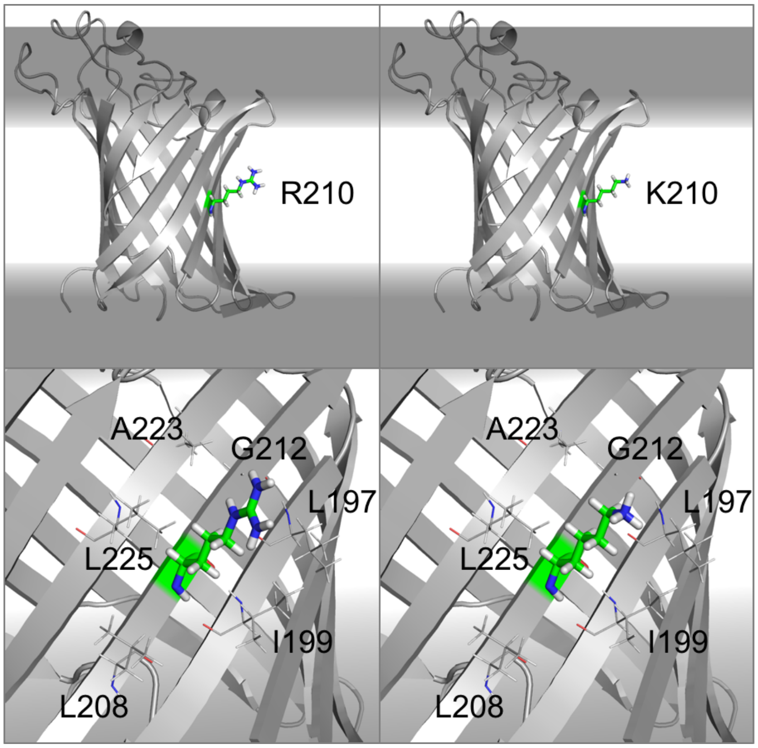

Fig. C: Close-up view of the arginine and lysine side chains and their neighboring residues

The amine charges at the end of the arginine side chain reach further towards the interface region than for lysine, therefore resulting in a smaller penalty of insertion.

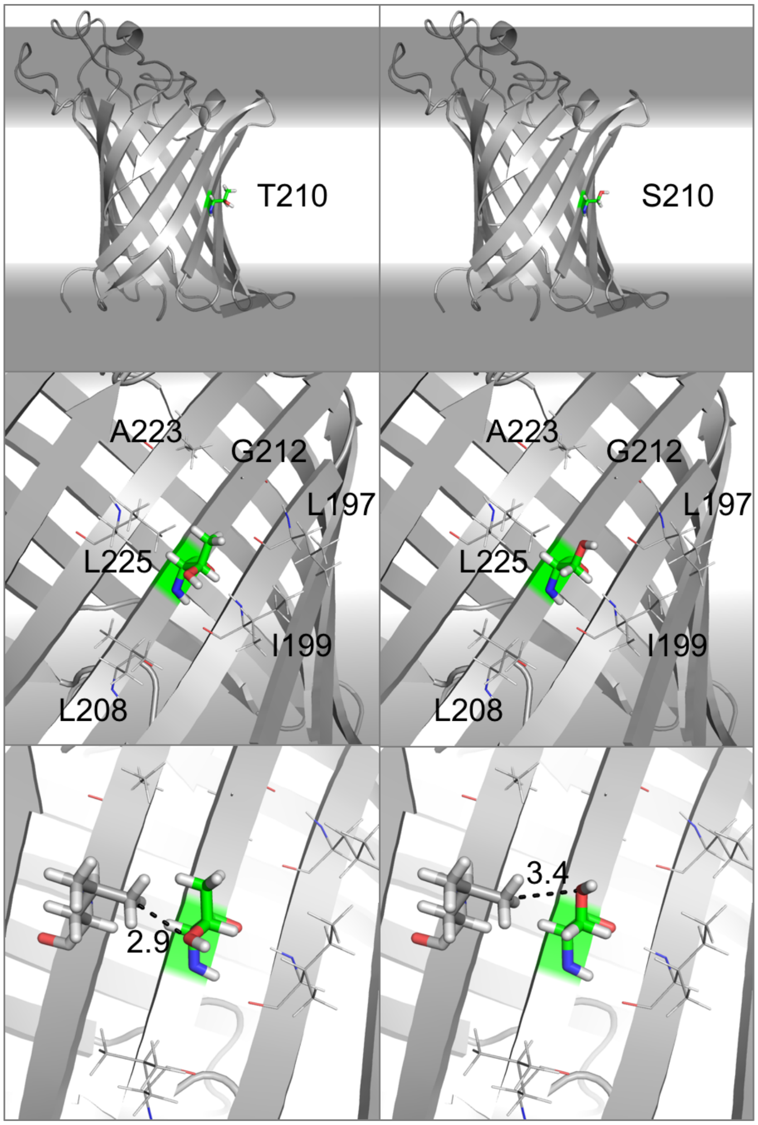

Fig. D: Close-up view of the threonine and serine side chains and their neighboring residues
The threonine hydroxyl group is closer to the neighboring leucine 225, leading to a larger van der Waals repulsive score and thus a larger $\Delta\Delta G$. The hydroxyl group in serine is further away from this leucine, influencing both the attractive and repulsive scores only marginally, leading to a smaller predicted $\Delta\Delta G$ compared to threonine.

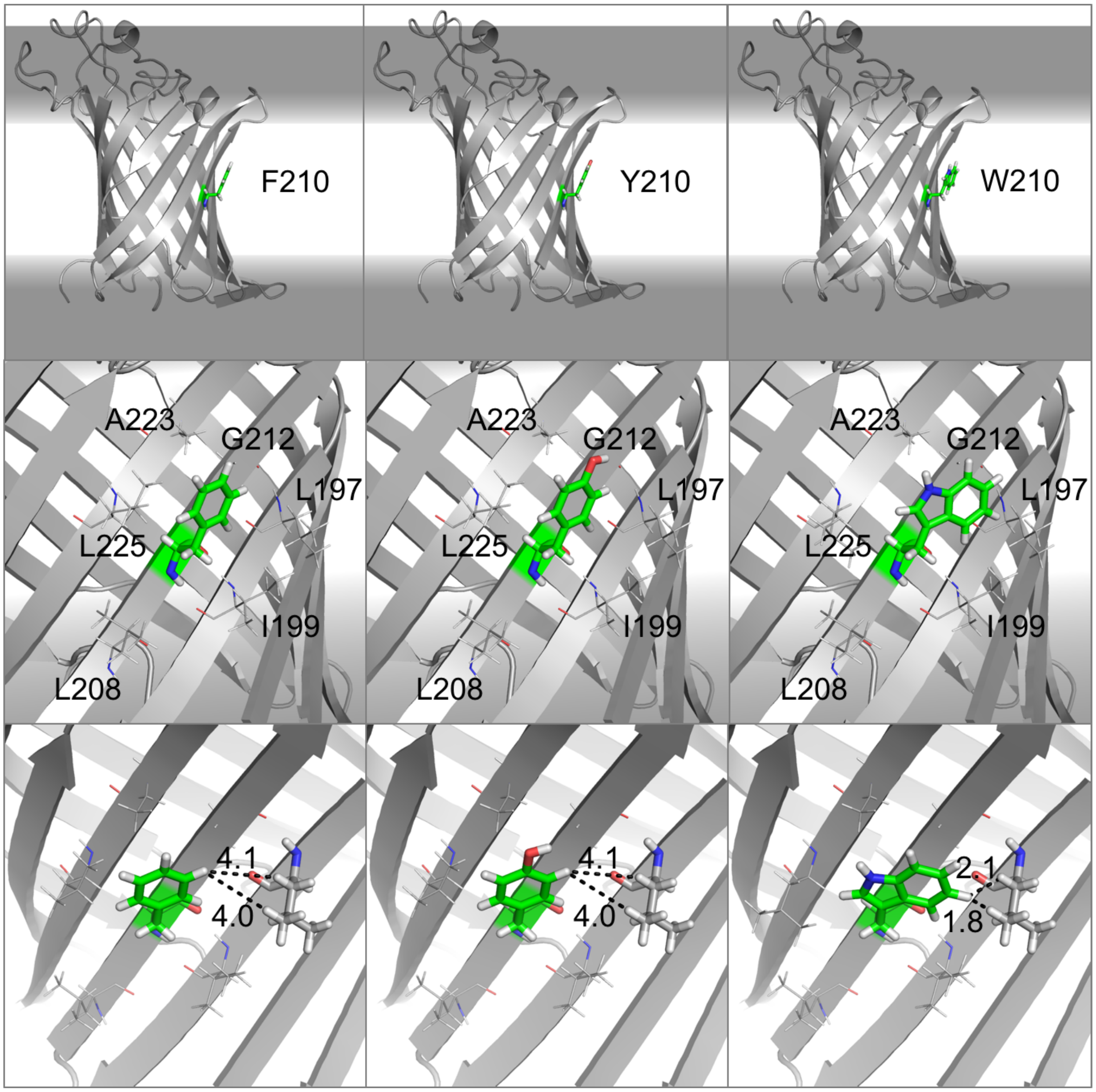

Fig. E: Close-up view of the aromatic side chains and their neighboring residues
Phenylalanine and tryptophan both have distances in the 4 Å range to the neighboring leucine 197. Tryptophan has smaller distances due to the additional benzene ring that clashes with the leucine and leads to larger differences in attractive and repulsive scores.

Table F: Contribution of individual Rosetta scores to predicted ΔΔ*G* values for mutations in OmpA

mutation	total	fa_atr	fa_rep	fa_intra_ rep	fa_elec	pro_close	fa_mpsolv	rama	fa_dun	p_aa_pp	ref	fa_mpenv	fa_mpenv_ smooth
W7A	0.137	9.398	-6.180	-0.025	-0.483	0.000	-1.138	0.184	-1.900	0.219	-0.750	-0.192	1.004
W15A	-43.030	6.988	-46.713	-0.023	0.032	0.000	-1.462	0.082	-2.421	-0.025	-0.750	0.041	1.221
W44A	3.681	7.000	-1.506	-0.028	0.476	0.000	-0.876	0.126	-1.695	0.240	-0.750	-0.333	1.027
W123A	5.646	7.353	-0.427	-0.026	0.122	0.000	-0.711	0.109	-1.110	0.160	-0.750	-0.187	1.113
Y30A	0.740	7.668	-4.088	-0.025	0.134	0.000	-0.891	0.240	-2.142	0.438	-0.350	-0.444	0.200
Y42A	0.937	4.312	-0.908	-0.027	0.698	0.000	-0.609	0.219	-2.788	0.443	-0.350	-0.309	0.256
Y109A	-2.684	4.681	-3.875	-0.033	0.371	0.000	-0.628	0.321	-3.341	0.382	-0.350	-0.481	0.269
Y121A	-0.005	4.913	-1.065	-0.024	0.239	0.000	-0.546	0.248	-3.764	0.322	-0.350	-0.422	0.444
Y134A	2.332	2.715	0.018	-0.017	0.043	0.000	0.042	0.242	-0.425	0.298	-0.350	-0.428	0.194
F38A	2.482	2.713	-0.089	-0.022	-0.088	0.000	-0.275	0.222	-1.267	0.289	-0.470	0.509	0.960
F103A	2.139	0.710	3.283	-0.031	0.010	0.000	-0.113	0.236	-3.063	0.334	-0.470	0.507	0.736
F136A	2.061	3.054	-0.089	-0.022	0.087	0.000	-0.280	-0.134	-1.660	-0.184	-0.470	0.511	1.248

Mutations are grouped by amino acid type (from top to bottom): tryptophan, tyrosine and phenylalanine. Positive values (red) indicate the native aromatic residue is favored and negative values (blue) indicate alanine is favored. In most cases, a loss of van der Waals attractive energy (fa_atr) is seen when aromatics are substituted with alanine. The knowledge-based membrane environment energies (fa_mpenv_smooth) also classifies mutation to alanine to be unfavorable, consistent with the published values. However, the Lazaridis membrane environment and solvation energies predict mutations to be favorable, indicating that improvements to the implicit membrane model are needed to accurately represent aromatics at the interface.

Detailed methods and command lines

ΔΔG values were predicted using the `predict_ddG.py` python script in the protocol capture in supporting information S2.

The following command line and options were used for prediction of ΔΔ*G* values for mutations in the protein OmpLA:

```
./predict_ddG.py
-in_pdb inputs/1qd6_tr_C.pdb                     # Input PDB file
-in_span inputs/1qd6_tr_C.span                   # Input spanfile
-out ddGs_ompLA.txt                              # Output filename with predicted ddGs
-res 181                                         # Pose residue number to mutate
-repack_radius 8.0                               # Repack all residues within X
                                                 # Angstroms of the mutant position
```

Using this command line, the script will predict the ΔΔ*G* values of mutation from alanine to all canonical residues.

Correlation plots were generated using the data from the output file containing predicted ΔΔ*G* values (`ddGs_ompLA.txt`). Tables describing the contribution of individual terms were created from the output score breakdown files.

The following command line and options were used for prediction of ΔΔ*G* values for mutations in the protein OmpA. Including the `–mut` option defines the specific mutant of interest:

```
./predict_ddG.py
-in_pdb inputs/1qjp_tr.pdb                       # Input PDB file
-in_span inputs/1qjp_tr.span                     # Input spanfile
-out ddGs_ompLA.txt                              # Output filename with predicted ddGs
-res 181                                         # Pose residue number to mutate
-mut A                                           # Mutate position to alanine
-repack_radius 8.0                               # Repack all residues within 8
                                                 # Angstroms of the mutant position
```

Correlation plots were generated using the data from the output file containing predicted ΔΔ*G* values. Tables describing contribution of individual terms were created from the output score breakdown files.

MPrelax: High-resolution membrane protein refinement

The MPrelax application described here simultaneously refines the structure and optimizes the membrane position and orientation.

Additional test cases

MPrelax was tested on a set of four membrane protein structures: meta-rhodopsin II (main text) and three additional cases described here. A diverse set of protein structures was selected to probe strengths and weaknesses in the refinement protocol. The cases are described in reference to Fig. F

(A) **Membrane methyltransferase,** monomer, crystal structure (PDB 4a2n) with five transmembrane helices. Three of five helices are significantly longer than the membrane thickness, suggesting a wider range of possible membrane embeddings along the z-axis.

(B) **Histidine kinase receptor QseC,** monomer, NMR structure (PDB 2kse) with two transmembrane helices.

(C) **Disulfide bonding protein B,** monomer, NMR structure (PDB 2leg) with four transmembrane helices. The predicted transmembrane spans are shorter than the hydrophobic thickness, increasing conformational space for the membrane embedding. The structure also contains a long loop region at the membrane interface.

Each protein structure was refined using the original membrane relax protocol described in Barth, 2007 [10] and the new MPrelax protocol to compare performance. 1000 refined models were generated for each simulation. The complete set of results is shown in Fig. F RosettaMP samples lower scoring models for meta-rhodopsin II (PDB 3pxo), membrane methyltransferase (PDB 4a2n) and histidine kinase receptor QseC (PDB 2kse). In contrast, the original RosettaMembrane protocol (Barth, 2007) leads to lower scores for disulfide bonding protein B (PDB 2leg). These preliminary data suggest that the minimization routine for optimizing the membrane position improves refinement of proteins that completely span the hydrophobic thickness of the membrane; however, requires improvement for proteins that do not fully span the membrane.

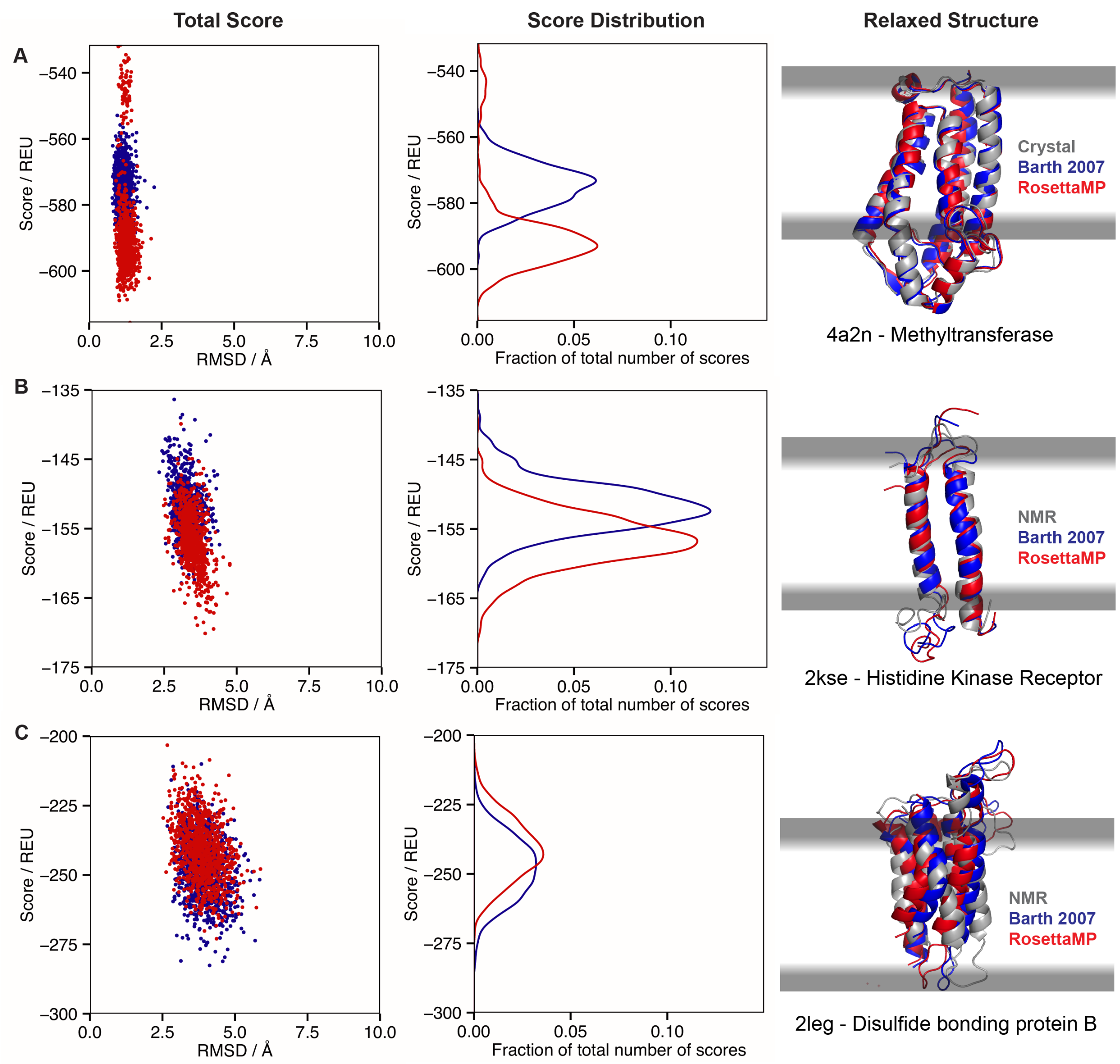

Fig. F: Additional test cases for membrane protein refinement
Left panel: Total score vs. RMSD plots for refined models. Center panel: Distribution of total Rosetta scores for refined models. Right panel: Lowest scoring refined models superimposed onto the native structure. Native shown in gray, models refined with new protocol shown in red, and models refined with original protocol shown in blue. (A) RosettaMP protocol samples lower scoring models of Methyltransferase (PDB 4a2n). (B) RosettaMP protocol samples lower scoring models of Histidine Kinase Receptor (PDB 2kse). (C) Barth 2007 protocol samples lower scoring models of Disulfide bonding protein B (PDB 2leg).

Origin of differences between RosettaMembrane (Barth, 2007) and RosettaMP refinement

To further investigate differences between the original RosettaMembrane (Barth, 2007) and RosettaMP relax protocols, the five lowest scoring models from both runs were compared using individual Rosetta energies, shown in Table G-J. In each table, scores are colored by column using a blue-to-red gradient, where blue is more favorable, and red is less favorable. Models in each category are listed from highest to lowest scoring model. An extended explanation for Tables G-J follows on p. 21.

Table G: Comparison of scores from five lowest scoring models of Metarhodopsin II (PDB ID 3pxo) refined with RosettaMembrane and RosettaMP refinement protocols

Method	total_score	fa_mpenv[1]	fa_mpenv_smooth[2]	fa_mpsolv	hbond_sr_bb	hbond_lr_bb	hbond_bb_sc	hbond_sc
Barth 2007	-964.05	-48.435	71.409	291.819	-139.899	-16.056	-71.493	-54.839
	-965.418	-47.48	71.388	291.731	-139.725	-13.135	-90.308	-44.144
	-966.71	-52.333	70.673	291.84	-143.675	-14.192	-75.912	-52.181
	-966.981	-50.734	71.909	289.014	-138.162	-16.878	-84.329	-44.371
	-970.462	-48.829	71.428	291.314	-140.099	-16.09	-81.123	-51.557
Rosetta MP	-988.055	-49.758	71.817	289.794	-161.451	-15.974	-71.885	-47.48
	-988.651	-51.192	71.41	292.234	-156.845	-16.001	-74.569	-59.633
	-988.913	-53.591	71.243	287.381	-160.683	-15.956	-74.905	-59.84
	-991.384	-48.841	70.976	292.413	-156.109	-15.974	-81.142	-59.486
	-992.678	-51.45	72.178	289.926	-152.173	-17.249	-72.552	-55.92

[1] Lazaridis membrane environment term
[2] Knowledge-based membrane environment term

Table H: Comparison of scores from five lowest scoring models of Methyltransferase (PDB ID 4a2n) refined with RosettaMembrane and RosettaMP refinement protocols

Method	total_score	fa_mpenv[1]	fa_mpenv_smooth[2]	fa_mpsolv	hbond_sr_bb	hbond_lr_bb	hbond_bb_sc	hbond_sc
Barth 2007	-591.527	-37.663	57.079	172.172	-78.092	-8.476	-30.302	-45.290
	-591.559	-38.811	57.315	171.505	-79.786	-9.665	-31.651	-37.632
	-595.133	-39.117	57.111	177.019	-78.955	-6.725	-33.640	-45.644
	-596.276	-39.079	57.132	171.872	-80.741	-9.009	-26.336	-44.236
	-596.528	-38.047	57.839	171.368	-78.674	-8.537	-32.578	-47.246
Rosetta MP	-607.405	-38.254	53.993	173.016	-87.645	-10.691	-28.264	-46.899
	-607.708	-40.795	55.644	173.922	-89.761	-13.157	-33.355	-41.864
	-608.727	-38.353	54.294	173.444	-88.168	-11.945	-32.086	-46.437
	-608.989	-39.871	55.898	171.130	-85.611	-12.990	-31.234	-44.253
	-615.630	-36.166	54.218	173.895	-91.970	-10.564	-31.084	-40.288

[1] Lazaridis membrane environment term
[2] Knowledge-based membrane environment term

Table I: Comparison of scores from five lowest scoring models of Histidine Kinase (PDB ID 2kse) refined with RosettaMembrane and RosettaMP refinement protocols

Method	total_score	fa_mpenv[1]	fa_mpenv_ smooth[2]	fa_mpsolv	hbond_sr_bb	hbond_lr_bb	hbond_bb_sc	hbond_sc
Barth 2007	-161.490	-12.048	16.287	48.832	-28.310	-1.656	-2.700	-4.120
	-161.570	-8.104	13.272	52.138	-29.310	-1.415	-8.441	-1.421
	-161.661	-10.063	18.737	47.361	-28.965	-1.543	-3.067	-3.261
	-162.368	-7.277	20.551	48.859	-28.771	-1.723	-2.082	-3.269
	-162.818	-10.700	13.160	50.194	-29.158	-0.393	-4.434	-2.809
RosettaMP	-167.881	-11.559	19.261	51.806	-31.505	-1.689	-6.977	-2.287
	-168.497	-11.231	19.345	50.358	-31.198	-1.202	-2.617	-4.623
	-168.923	-12.848	19.522	50.661	-30.512	-1.666	-3.394	-5.081
	-169.485	-11.690	17.061	50.317	-32.151	-1.176	-6.304	-2.974
	-170.125	-11.426	17.607	52.158	-32.179	-1.284	-7.343	-3.936

[1] Lazaridis membrane environment term

[2] Knowledge-based membrane environment term

Table J: Comparison of scores from five lowest scoring models of Disulfide Bonding Protein B (PDB ID 2leg) refined with RosettaMembrane and RosettaMP refinement protocols

Method	total_score	fa_mpenv[1]	fa_mpenv_ smooth[2]	fa_mpsolv	hbond_sr_bb	hbond_lr_bb	hbond_bb_sc	hbond_sc
Barth 2007	-277.654	-3.938	28.849	104.883	-50.970	-1.975	-14.204	-9.331
	-279.620	0.068	29.979	108.801	-52.058	-4.528	-11.897	-18.432
	-280.941	-6.529	29.187	103.456	-48.043	-3.828	-18.123	-10.515
	-281.118	-6.005	31.270	105.172	-54.976	-2.539	-13.957	-16.818
	-282.608	-5.117	30.829	107.877	-53.210	-3.058	-9.321	-10.200
RosettaMP	-265.550	8.466	-0.932	131.532	-53.506	-3.153	-12.791	-12.967
	-266.446	12.472	-1.513	130.183	-55.243	-1.826	-10.518	-17.907
	-268.315	13.116	-1.197	128.330	-59.892	-2.602	-17.067	-11.202
	-270.184	10.643	-1.249	133.923	-55.832	-5.819	-12.482	-14.585
	-273.052	14.246	-0.770	131.570	-56.804	-3.899	-9.697	-12.050

[1] Lazaridis membrane environment term

[2] Knowledge-based membrane environment term

Rosetta scores for the top five scoring models from RosettaMembrane (Barth, 2007) and RosettaMP relax are decomposed into individual weighted score terms (weights in Table D). Both protocols sample models within similar RMSD ranges (Fig. G) suggesting differences are attributed to different positions and orientations (embedding) of proteins in the membrane. In Tables G-J we show all score terms dependent on membrane embedding: Lazaridis membrane environment (fa_mpenv), Lazaridis membrane solvation (fa_mpsolv), knowledge-based membrane environment (fa_mpenv_smooth), and hydrogen bonding energies (hbond_sc, hbond_bb_sc, hbond_lr_bb, hbond_sr_bb).

(A) **Table G:** Comparison of meta-rhodopsin II (PDB 3pxo) refined models. Scores for short-range backbone-backbone (hbond_sr_bb) and side chain – side chain (hbond_sc) hydrogen bonds are consistently lower in RosettaMP models. Since both models contain similar numbers of hydrogen bonds, lower scores are attributed to improved positioning of hydrogen bonds in the membrane.

(B) **Table H:** Comparison of membrane methyltransferase (PDB 4a2n) refined models. Scores for short-range and long-range backbone-backbone hydrogen bonds (hbond_sr_bb and hbond_lr_bb) are consistently lower for RosettaMP models. Since both models contain similar numbers of hydrogen bonds, lower scores are attributed to improved positioning of hydrogen bonds in the membrane. RosettaMP models also have lower knowledge-based membrane environment scores, further indicating membrane embedding is improved in these models.

(C) **Table I:** Comparison of histidine kinase receptor QseC (PDB 2kse) models. Scores for short-range backbone-backbone hydrogen bonds (hbond_sr_bb) are consistently lower for RosettaMP models. Both models contain similar numbers of hydrogen bonds, indicating lower scores are attributed to improved positioning of hydrogen bonds in the membrane.

(D) **Table J:** Comparison of Disulfide bond protein B (PDB 2leg) models. Scores for Lazaridis membrane environment and solvation energies are consistently higher in models refined by RosettaMP. This protein does not fully span the membrane, complicating the task of simultaneously embedding and refining a protein with a moveable membrane. These data suggest the fixed membrane used in Barth, 2007 is still better for such cases, pointing to needed improvements in the minimization routine used by RosettaMP.

Detailed methods and command lines

Refinement with the original membrane relax application as described in [10] was performed with the following application and options. Here, 3PXO is used as an example protein:

```
Rosetta/main/source/bin/relax.linuxgccrelease
-in:file:s 3PXO_native.pdb                        # Input PDB file
-in:file:spanfile 3PXO.span                       # Input spanfile
-nstruct 1000                                     # Generate 1000 models
-relax:fast                                       # Use FastRelax protocol
-score:weights membrane_highres_Menv_smooth       # Use hi-res membrane score function
-membrane:fixed_membrane                          # Keep membrane position fixed
-membrane:Mhbond_depth true                       # Turn on membrane hydrogen bonding
```

Note, the weights file, `membrane_highres_Menv_smooth` is the original implementation of the high resolution membrane score function [10].

To compute the RMSD between the native and refined models, we used the `score_jd2` application with the following options:

```
Rosetta/main/source/bin/score_jd2.linuxgccrelease
-in:file:l 3PXO_models_list                       # List of refined models
-in:file:native 3PXO_native.pdb                   # Native structure for RMSD calculation
-in:file:spanfile 3PXO.span                       # Input spanfile
-score:weights membrane_highres_Menv_smooth       # Use hi-res membrane score function
-membrane:fixed_membrane                          # Keep membrane position fixed
-membrane:Mhbond_depth true                       # Turn on membrane hydrogen bonding
```

The new membrane relax application is captured in the following RosettaScript:

```
<ROSETTASCRIPTS>
  <SCOREFXNS>
    <memb_hires weights=mpframework_smooth_fa_2012 />
    </SCOREFXNS>
  <MOVERS>
    <AddMembraneMover name=add_memb/>
    <MembranePositionFromTopologyMover name=init_pos/>
    <FastRelax name=fast_relax scorefxn=memb_hires repeats=8/>
    </MOVERS>
  <PROTOCOLS>
    <Add mover=add_memb/>
    <Add mover=init_pos/>
    <Add mover=fast_relax/>
    </PROTOCOLS>
</ROSETTASCRIPTS>
```

The following application and options were used to generate 1000 refined models:

```
Rosetta/main/source/bin/rosetta_scripts.linuxgccrelease
-parser:protocol membrane_relax.xml               # Path to Rosetta script (see above)
-in:file:s 3PXO_tr_native.pdb                     # Input PDB structure
-nstruct 1000                                     # Generate 1000 models
-mp:setup:spanfiles 3PX0.span                     # Input spanfile
-mp:scoring:hbond                                 # Turn on membrane hydrogen bonding
-relax:jump_move true                             # Allow jumps to move during relax
-packing:pack_missing_sidechains 0                # Wait to pack sidechains until
                                                  # membrane is present
```

Because the Rosetta Relax will not automatically compute the RMSD between the native structure and refined model, the following application and options were used to compute the RMSD between the native and refined models:

```
Rosetta/main/source/bin/score_jd2.linuxgccrelease
-in:file:s 3PXO_models_list                   # List of refined models
-in:file:native 3PXO_native.pdb               # Native structure for RMSD calculation
-score:weights mpframework_smooth_fa_2012     # Use hi-res membrane score function
-in:membrane                                  # Input is a membrane protein
-mp:setup:membrane_rsd 100                    # Read membrane residue at position 100
-mp:setup:spanfiles 3PXO.span                 # Input spanfile
-mp:scoring:hbond                             # Turn on membrane hydrogen bonding
-packing:pack_missing_sidechains 0            # Wait to pack sidechains until
                                              # membrane is present
```

Score vs. RMSD plots were generated using the output score file from the re-scoring step. RMSDs are calculated with respect to the backbone atoms of the native structure. Score density plots were also generated from this output file.

Refinement of Metarhodopsin II (PDB 3pxo) with Retinal

A structure of meta-rhodopsin II crystallized in complex with retinal is available (RET; PDB 3pxo) [21]. To test the performance of MPrelax on this protein-ligand complex, we created an additional mover that accommodates the ligand in the membrane FoldTree. This mover, `AddMPLigandMover`, attaches the ligand to the protein by jump, preserving the membrane jump. All stages of refinement are identical except that no rotamers are sampled for the ligand.

In the presence of Retinal, MPrelax samples a broader range of protein conformations than without it (Fig. G); between 1.0-5.0 Å RMSD to the crystal structure, compared to only 1.0-2.5 Å without the ligand. The lowest scoring refined model in the presence of the ligand has a smaller RMSD to the crystal structure, at 1.3 Å compared to 1.6 Å without the ligand. Because the original membrane relax protocol [10] does not accommodate ligands, we were unable to compare the performance of these methods. While this preliminary data is encouraging, some protocol enhancements that need to be explored include combining RosettaLigand [22] with RosettaMP to sample flexible ligand conformations, and benchmarking a score function in the membrane environment that includes ligands.

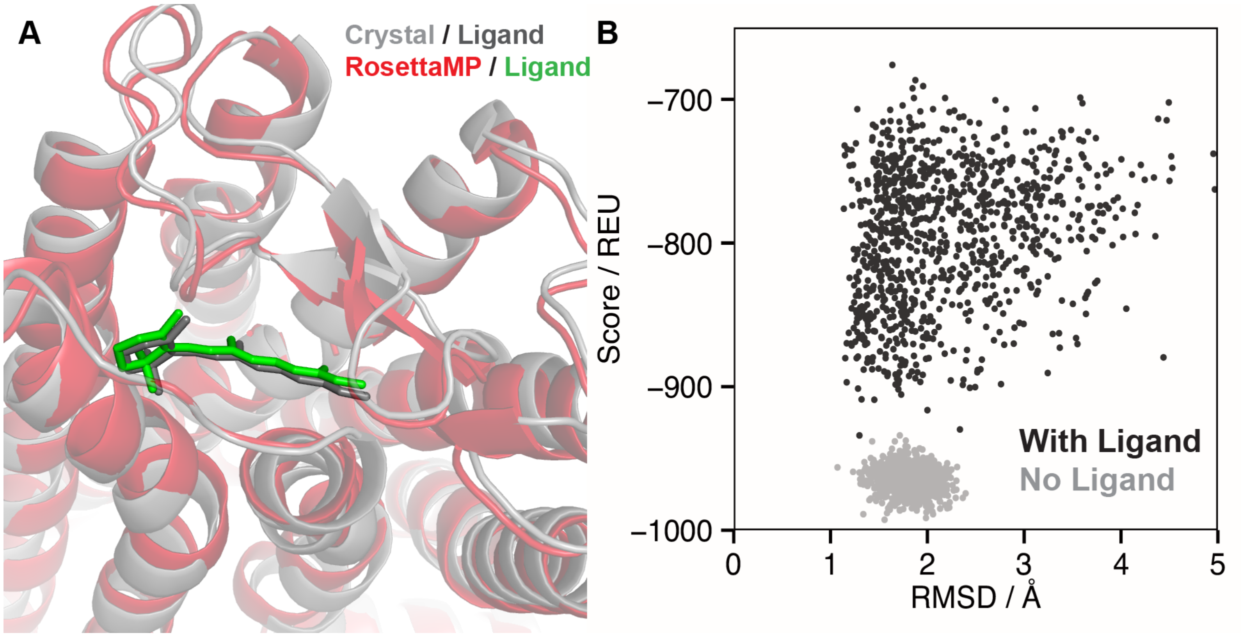

Fig. G: Refinement of metarhodopsin II in the presence of retinal
(A) Structure of the lowest scoring refined model using the Rosetta MPrelax method (red) superimposed onto the crystal structure (PDB 3pxo; gray). Retinal in the refined model is shown in green, while in the crystal structure it is shown in dark gray. (B) Total score vs. RMSD plots from refinement. Models refined without the ligand (as shown in Fig. 6 in the main text) are shown in light gray, and models refined with the ligand are shown in black.

Detailed methods and command lines

The Rosetta script for refinement of a protein-ligand complex is presented below. The additional mover, `AddMPLigandMover`, accommodates the ligand in the membrane FoldTree and requires two arguments: (1) sequence position of the ligand in the PDB file, and (2) a residue in the binding pocket close to the ligand.

```
<ROSETTASCRIPTS>
  <SCOREFXNS>
    <memb_hires weights=mpframework_smooth_fa_2012 />
  </SCOREFXNS>
  <MOVERS>
    <AddMembraneMover name=add_memb/>
    <MembranePositionFromTopologyMover name=init_pos/>
    <AddMPLigandMover name=add_ligand ligand_seqpos=327 closest_rsd=118 />
    <FastRelax name=fast_relax scorefxn=memb_hires repeats=8/>
  </MOVERS>
  <PROTOCOLS>
    <Add mover=add_memb/>
    <Add mover=init_pos/>
    <Add mover=add_ligand/>
    <Add mover=fast_relax/>
  </PROTOCOLS>
</ROSETTASCRIPTS>
```

The Rosetta ligand parameter file (RET.params) for retinal was prepared as described in [22]. MPrelax uses the following application and options; 1000 models were generated:

```
Rosetta/main/source/bin/rosetta_scripts.linuxgccrelease
-parser:protocol membrane_relax.xml           # Path to Rosetta script (see above)
-in:file:s 3PXO_tr_native.pdb                 # Input PDB structure
-in:extra_res_fa RET.params                   # Rosetta parameter file for retinal
-nstruct 1000                                 # Generate 1000 models
-mp:setup:spanfiles 3PX0.span                 # Input spanfile
-mp:scoring:hbond                             # Turn on membrane hydrogen bonding
-relax:jump_move true                         # Allow jumps to move during relax
-packing:pack_missing_sidechains 0            # Wait to pack sidechains until
                                              # membrane is present
```

Because MPrelax does not automatically compute the RMSD between the native structure and refined model, the following application and options were used to compute the RMSD between the native and refined models:

```
Rosetta/main/source/bin/score_jd2.linuxgccrelease
-in:file:s 3PXO_models_list                   # List of refined models
-in:extra_res_fa RET.params                   # Rosetta parameter file for retinal
-in:file:native 3PXO_native.pdb               # Native structure for RMSD calculation
-score:weights mpframework_smooth_fa_2012     # Use hi-res membrane score function
-in:membrane                                  # Input is a membrane protein
-mp:setup:membrane_rsd 328                    # Read membrane residue at position 328
-mp:setup:spanfiles 3PXO.span                 # Input spanfile
-mp:scoring:hbond                             # Turn on membrane hydrogen bonding
-packing:pack_missing_sidechains 0            # Wait to pack sidechains until
                                              # membrane is present
```

Score vs. RMSD plots were generated using the output score file from the re-scoring step. RMSDs are calculated with respect to the backbone atoms of the native structure.

MPdock: Membrane protein-protein docking

This protocol combines the RosettaDock algorithm with the membrane energy function and adapted initial sampling to model the structure of protein-protein complexes in the membrane.

Additional test cases

The membrane protein-protein docking protocol was tested on a set of 5 membrane protein complexes: Vitamin B12 importer (PDB 2qi9) discussed in the main text and four cases discussed below. For each protein, 10 locally refined models and 1000 docked models were generated during the simulation. Results for each protein are shown in Fig. H

(A) **Glycophorin A**, homodimer, NMR structure (PDB 1afo) with one transmembrane helix per subunit. Docking partners are chain A and B. Since this is an NMR structure and Rosetta's energy function is parameterized on crystal structures, it is expected that the lowest scoring models have higher RMSDs than models compared to the crystal structure. MPdock samples two conformations: models around 1 Å and 5 Å RMSD, the latter with more favorable interface scores, suggesting needed improvements to the scorefunction.

(B) **Putative metal-chelate type ABC transporter**, crystal structure (PDB 2nq2) with nine transmembrane helices for each chain A and B. Chains C and D are soluble domains. Docking partners are chains AC and BD. MPdock samples models as low as 0.3 Å from the crystal structure and recovers up to 96 % of contacts. Several models are excluded from Fig. HB due to high interface scores, suggesting further work is needed to accommodate both transmembrane and soluble domain interfaces during sampling.

(C) **Integrin αIIb-β3 heterodimer**, NMR structure (PDB 2k9j) with one transmembrane helix per subunit. Docking partners are chains A and B. MPdock samples models as low as 1.8 Å from the crystal structure ad recovers up to 75% of native contacts.

(D) The transmembrane domain of the homodimeric **MetNI methionine ABC transporter** crystal structure (PDB 3tui) with five transmembrane helices per subunit. Docking partners are chains A and B. Interestingly, MPdock samples two conformations for this protein. The protein exhibits an open (claw-like) conformation with a small interface and undergoes a conformational change for transport, with the closed conformation having an increased number of contacts and a larger interface. Here, both conformations are sampled (funnels around 0 Å and 10 Å) but scores the one with the larger interface more preferably, due to the higher number of contacts.

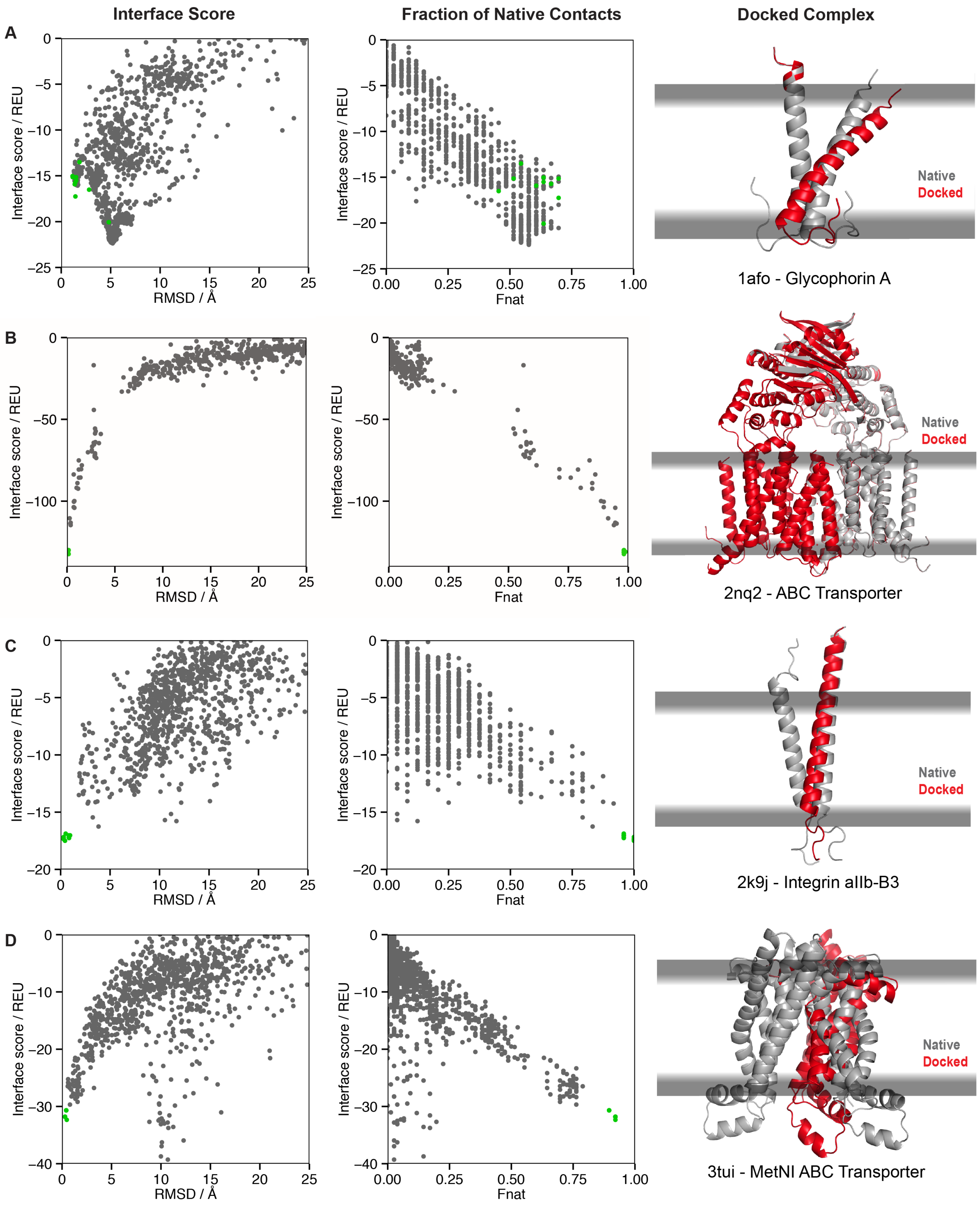

Fig. H: Additional test cases for intra-membrane protein-protein docking
Left panel: Interface score vs. RMSD plots. Center panel: Interface score vs. Fraction of native contacts. Gray points represent docked models and the green dots represent 10 models created by refining the crystal structure. Right panel: Lowest scoring docked model (red) superimposed onto the native. The subunit colored in gray remains fixed throughout the protocol.

Detailed methods and command lines

Additional model preparation: To allow the use of the two-body docking algorithm, chains from the extra-membrane region were removed, as were additional chains that would interfere with the docking algorithm.

The prepack protocol was run with the following application and options. Here, 1afo is used as an example protein:

```
Rosetta/main/source/bin/docking_prepack_protocol.linuxgccrelease
-in:file:s 1AFO_AB.pdb                       # Input PDB containing both partners
-nstruct 10                                  # Generate 10 models
-score:weights mpframework_docking_fa_2015.wts # Use mpdocking score function
-mp:setup:spanfiles 1AFO_AB.span             # Input spanfile
-mp:scoring:hbond                            # Turn on membrane hydrogen bonding
-packing:pack_missing_sidechains 0           # Wait to pack sidechains until
                                             # membrane is present
```

10 structures were generated using the prepack protocol and the lowest-scoring model (`1AFO_AB_ppk.pdb`) by total Rosetta score was chosen as input to the membrane protein-protein docking protocol. The following options were used:

```
Rosetta/main/source/bin/mpdocking.linuxgccrelease
-in:file:s 1AFO_AB_ppk.pdb                   # Pre-packed input structure
-in:file:native 1AFO_AB.pdb                  # Native structure for RMSD calculation
-nstruct 1000                                # Generate 1000 models
-score:weights mpframework_docking_fa_2015.wts # Score function
-mp:setup:spanfiles 1AFO_AB.span             # Input spanfile
-mp:scoring:hbond                            # Turn on membrane hydrogen bonding
-docking:partners A_B                        # Partners to dock
-docking:dock_pert 3 8                       # Magnitude of perturbation
-packing:pack_missing_sidechains 0           # Wait to pack sidechains until
                                             # membrane is present
```

1000 models were created with a default docking perturbation of 3 Å translation and 8 degrees rotation.

Additionally, the ‘native’ structures were refined to compare both sampling and scoring of the membrane protein-protein docking protocol to these models. 10 models were created using the options:

```
Rosetta/main/source/bin/mpdocking.linuxgccrelease
-in:file:s 1AFO_AB.pdb                       # Input PDB file
-in:file:native 1AFO_AB.pdb                  # Native structure for RMSD calculation
-nstruct 10                                  # Generate 10 models
-mp:setup:spanfiles 1AFO_AB.span             # Input spanfile
-score:weights mpframework_docking_fa_2015.wts # Use mpdocking score function
-mp:scoring:hbond                            # Turn on membrane hydrogen bonding
-docking:partners A_B                        # Partners to dock
-docking_local_refine                        # Only do a local refinement
-packing:pack_missing_sidechains 0           # Wait to pack sidechains until
                                             # membrane is present
```

Score vs. RMSD plots were generated from the output score files. RMSDs were ‘ligand’ RMSDs as computed in the third column in the score file, which were computed only over the movable partner in the complex.

Application MPsymdock: Assembly of symmetric membrane protein complexes

The MPsymdock protocol combines the symmetric docking protocol (RosettaSymDock) with RosettaMP to predict structures of symmetric complexes in the membrane environment.

Additional test cases

MPsymdock protocol was tested on a set of four membrane protein complexes with C_n symmetry, where n is the number of subunits in the complex: the KcsA Potassium channel (PDB 1bl8) and three additional cases discussed here. Each native protein complex was first refined using the membrane relax protocol. The lowest scoring refined native was then setup for assembly and 1000 docked models were generated.

(A) **Glycophorin A,** homo-dimer, NMR structure (PDB 1afo). For the starting structure, we used model 1 of the NMR structure from the PDBTM [15]. Symmetric assembly of this dimer, which we also attempted in asymmetric docking, creates low-energy structures with similar helix crossing angles as in the experimental structure. The RMSD of this structure is 6 Å, due to deviations in the comparison of the extra-membrane loops, which also complicate the detection of symmetry in generating the starting structure. The full set of 20 models in the NMR structure show broad diversity in the tail location, indicating that the NMR constraints do not fully specify the position.

(B) **Human Leukotriene C4 Synthase**, homo-trimer, crystal structure (PDB 2uuh). MPsymdock is not able to find the correct interface; instead the individual subunits rotate by about 90° along the membrane normal. In contrast to the native structure, the extra-membrane helices in the model are bent towards each other with steric clashes preventing the formation of a native-like interface.

(C) **Mechanosensitive channel of large conductance (MscL)**, homo-pentamer, crystal structure (PDB 2oar). The helices in each subunit form a claw-like interface with the neighboring subunits (i.e. a domain swap). These intertwined interfaces are challenging to form given the restricted rotation in symmetry. In practice, clashes in the model force the docking algorithm to separate the subunits, away from the experimentally determined conformation.

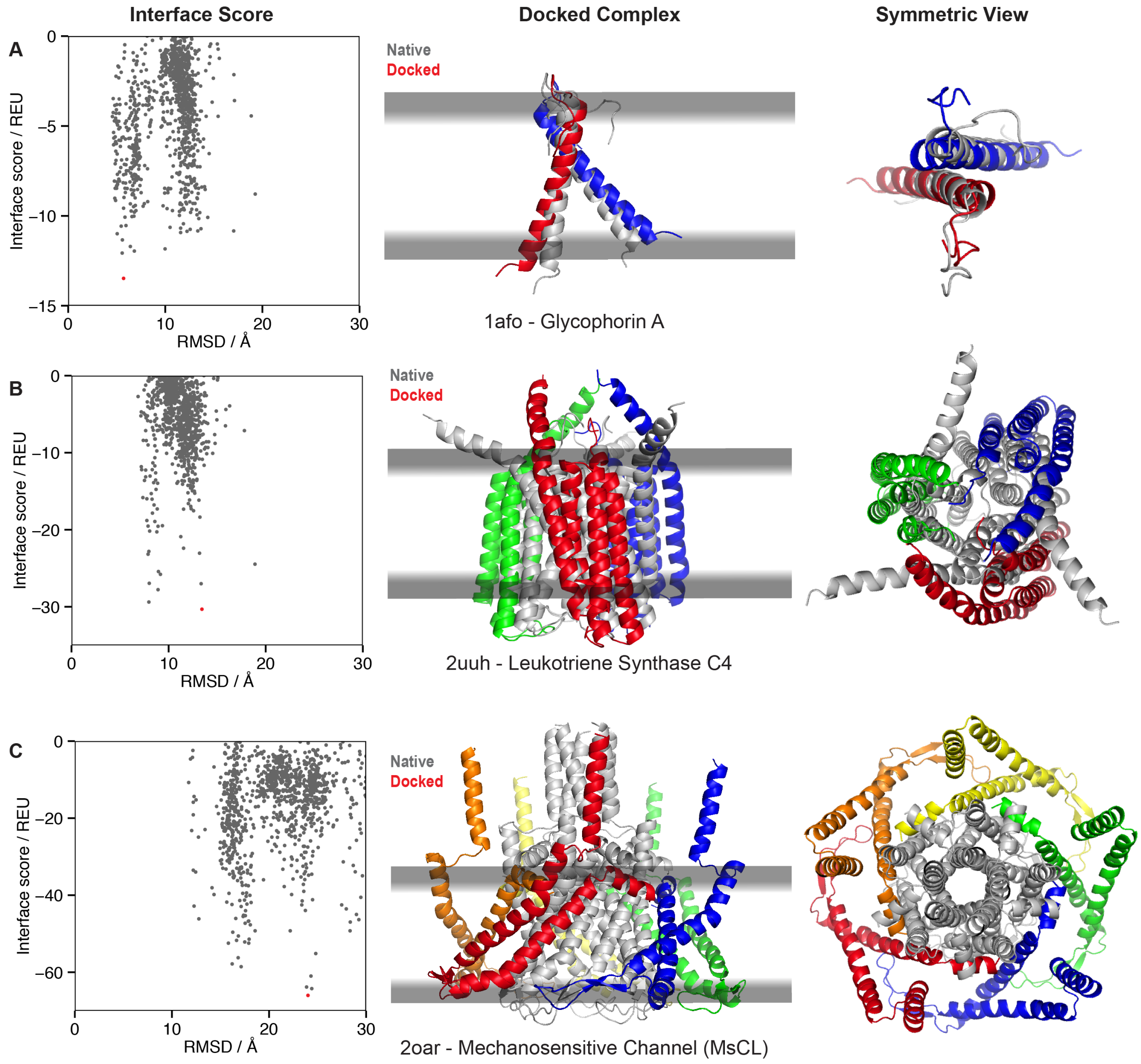

Fig. I: Test cases for membrane protein symmetric docking application
Left panel: Interface score vs. RMSD plots. Lowest interface scoring complex shown in red. Center panel: Lowest interface scoring complex (multi-color) superimposed onto the native (gray). Right panel: Top view of docked models superimposed onto native, displaying arrangement of symmetric subunits around the membrane normal axis. Each model is discussed on p. 29.

Detailed methods and command lines

To resolve initial clashes in the crystal structure, the native was refined using the MPrelax application described above. The following application and flags were used. Here, 1afo is used as an example:

```
Rosetta/main/source/bin/rosetta_scripts.linuxgccrelease
-parser:protocol membrane_relax.xml         # Path to Rosetta script (above)
-in:file:s 1afo_native.pdb                  # Input PDB file
-nstruct 10                                 # Generate 10 models
-mp:setup:spanfiles 1afo.span               # Input spanfile
-mp:scoring:hbond                           # Turn on membrane hydrogen bonding
-relax:jump_move true                       # Allow jumps to move during relax
-packing:pack_missing_sidechains 0          # Wait to pack sidechains until
                                            # membrane is present
```

10 refined native complexes were generated and the model with the lowest total Rosetta score was used as the starting structure for symmetric docking. From this starting structure, a symmetry definition file describing the arrangement of subunits around the C symmetry axis and input structure containing the asymmetric subunit was generated with the following script and options:

```
Rosetta/main/source/src/apps/public/symmetry/make_symmdef_file.pl > 1afo.c2.symm
-p 1afo_native.pdb                          # Refined input symmetric complex
-a A                                        # Chain to use as the master subunit
-b B:2                                      # 1st child subunit (B) and number of
                                            # subunits in the symmetric complex (2)
```

It is important to note that this script requires all chains be of equal length. The output file contains the symmetry definition required for symmetric modeling in Rosetta.

Because the starting structure was already transformed into the membrane coordinate frame, spanfiles were generated using the `span_from_pdb` application described below. Predicted TM spans are only provided as input for the asymmetric starting unit.

The following application and flags were used to generate models of the symmetric complex:

```
Rosetta/main/source/bin/membrane_symdocking.linuxgccrelease
-in:file:s 1afo_input.pdb                   # Asymmetric input structure
-in:file:native 1afo_tr_native.pdb          # Native structure for RMSD calculation
-nstruct 1000                               # Generate 1000 models
-mp:setup:spanfiles 1afo.span               # TM spans for asymm unit
-mp:scoring:hbond                           # Turn on membrane hydrogen bonding
-symmetry:symmetry_definition 1afo.c2.symm  # Symmetry definition file
-symmetry:initialize_rigid_body_dofs        # Sample configurations during assembly
-packing:pack_missing_sidechains 0          # Wait to pack sidechains until
                                            # membrane is present
-docking:dock_lowres_filter 5.0 10.0        # Change filters required for a low
                                            # resolution model to advance to
                                            # high-res stage. Vdw score < 5.0 and
                                            # interchain contact score < 10.0
```

Score vs. RMSD plots were generated from the output score file. RMSDs of the assembled complex are computed with respect to the backbone atoms of the native structure.

Membrane visualization

The PyMOL viewer extension for membrane proteins can be used with any Rosetta modeling application. In addition, we provide a standalone application useful for visualizing sets of output Rosetta models. This application, `view_membrane_protein`, will read an input PDB file or list of PDBs, initialize an implicit membrane at the default or user-specified position, and display the structures with the membrane planes in PyMOL.

An example of this visualization is shown in Fig. J.

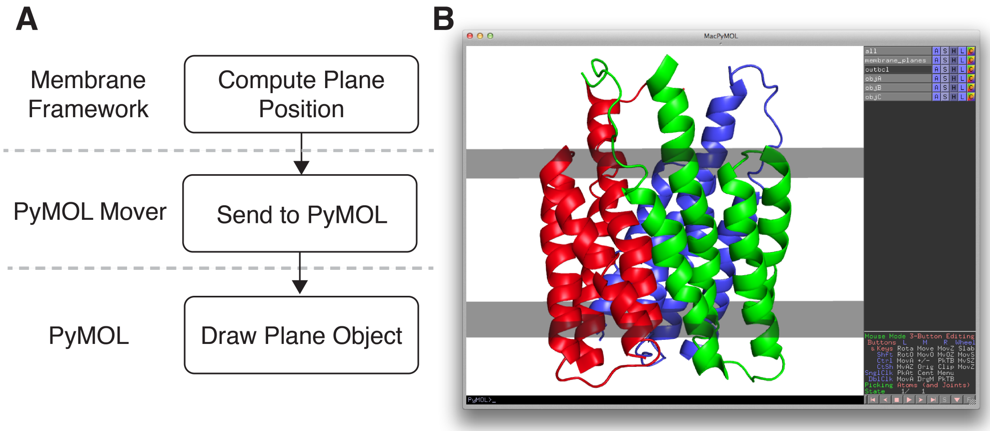

Fig. J: Membrane protein visualization using the PyMOL viewer
The membrane position and geometry can be visualized during a simulation or to analyze models using the PyMOL viewer in Rosetta. (A) This application computes the position of two parallel planes describing the membrane and sends these coordinates to PyMOL. PyMOL then uses compiled graphics objects to display the planes. (B) Example visualization of membrane planes during a simulation.

Detailed command lines

The following application and options can be used for standalone membrane protein visualization. Here, 1afo is used as an example protein with `1afo_list` being a file containing all models for visualization. This application recognizes the membrane residue in the output model PDB file unless center and normal are specified by the user as described below.

```
Rosetta/main/source/bin/view_membrane_protein.linuxgccrelease
-in:file:l 1afo_list                          # List of input models
-mp:setup:spanfiles 1AFO_AB.span              # Input spanfile
-mp:setup:center 0 0 0                        # (Optional) Specify membrane center
-mp:setup:normal 0 0 1                        # (Optional) Specify membrane normal
```

To visualize a simulation, start a new PyMOL session. In the PyMOL window, initialize the PyMOLMover by running the script `PyMOLPyrosettaServer.py`. A message will appear in the PyMOL Terminal indicating the server has been initialized successfully. Finally, from the regular terminal, run the standalone Rosetta application with the flags above.

Application MPspanfrompdb: Calculate transmembrane spans from structure

For known structures that are already transformed into a membrane coordinate frame, the protein coordinates in the PDB and the membrane thickness can be used to compute transmembrane spanning regions. Start and end residue numbers describing the transmembrane span are then output in a span file, an input required for all Rosetta membrane framework applications.

Detailed methods and command lines

Here, 1afo is used as an example protein.

```
Rosetta/main/source/bin/span_from_pdb.linuxgccrelease
-in:file:s 1afo_tr.pdb                        # Input PDB file
```

The output file format contains the number of transmembrane spans, total number of residues described by the topology, direction of the topology (N-terminus to C-terminus) and residue numbers describing start and end positions of individual transmembrane spanning regions.

```
Rosetta-generated spanfile from SpanningTopology object
2 80
antiparallel
n2c
     15   31
     55   73
```

References

1. Webb B, Lasker K, Schneidman-Duhovny D, Tjloe E, Phillips, Joong Kim S, et al. Modeling of Proteins and Their Assemblies with the Integrative Modeling Platform. Humana Press; 2011. Available: http://link.springer.com/protocol/10.1007/978-1-61779-276-2_19

2. Pettersen EF, Goddard TD, Huang CC, Couch GS, Greenblatt DM, Meng EC, et al. UCSF Chimera—A visualization system for exploratory research and analysis. J Comput Chem. 2004;25: 1605–1612. doi:10.1002/jcc.20084

3. Cock PJA, Antao T, Chang JT, Chapman BA, Cox CJ, Dalke A, et al. Biopython: freely available Python tools for computational molecular biology and. Bioinformatics. 2009;25: 1422–1423. doi:10.1093/bioinformatics/btp163

4. Adams PD, Afonine PV, Bunkóczi G, Chen VB, Davis IW, Echols N, et al. PHENIX : a comprehensive Python-based system for macromolecular structure solution. Acta Crystallogr D Biol Crystallogr. 2010;66: 213–221. doi:10.1107/S0907444909052925

5. Das R, Baker D. Macromolecular modeling with rosetta. Annu Rev Biochem. 2008;77: 363–382. doi:10.1146/annurev.biochem.77.062906.171838

6. Holland IM, Lieberherr KJ. Object-oriented Design. ACM Comput Surv. 1996;28: 273–275. doi:10.1145/234313.234421

7. Leaver-Fay A, Tyka M, Lewis SM, Lange OF, Thompson J, Jacak R, et al. Rosetta3: An Object-Oriented Software Suite for the Simulation and Design of Macromolecules. Michael L. Johnson and Ludwig Brand, editor. Methods Enzymol. 2011;Volume 487: 545–574.

8. Drew K, Renfrew PD, Craven TW, Butterfoss GL, Chou F-C, Lyskov S, et al. Adding diverse noncanonical backbones to rosetta: enabling peptidomimetic design. PloS One. 2013;8: e67051. doi:10.1371/journal.pone.0067051

9. Yarov-Yarovoy V, Schonbrun J, Baker D. Multipass membrane protein structure prediction using Rosetta. Proteins Struct Funct Bioinforma. 2006;62: 1010–1025. doi:10.1002/prot.20817

10. Barth P, Schonbrun J, Baker D. Toward high-resolution prediction and design of transmembrane helical protein structures. Proc Natl Acad Sci. 2007;104: 15682–15687. doi:10.1073/pnas.0702515104

11. Yarov-Yarovoy V, DeCaen PG, Westenbroek RE, Pan C-Y, Scheuer T, Baker D, et al. Structural basis for gating charge movement in the voltage sensor of a sodium channel. Proc Natl Acad Sci. 2012;109: E93–E102. doi:10.1073/pnas.1118434109

12. Lazaridis T. Effective energy function for proteins in lipid membranes. Proteins. 2003;52: 176–192. doi:10.1002/prot.10410

13. Rohl CA, Strauss CEM, Misura KMS, Baker D. Protein Structure Prediction Using Rosetta. In: Ludwig Brand and Michael L. Johnson, editor. Methods in Enzymology. Academic Press; 2004. pp. 66–93. Available: http://www.sciencedirect.com/science/article/pii/S0076687904830040

14. Kortemme T, Morozov AV, Baker D. An orientation-dependent hydrogen bonding potential improves prediction of specificity and structure for proteins and protein-protein complexes. J Mol Biol. 2003;326: 1239–1259.

15. Kozma D, Simon I, Tusnády GE. PDBTM: Protein Data Bank of transmembrane proteins after 8 years. Nucleic Acids Res. 2012; gks1169. doi:10.1093/nar/gks1169

16. Kellogg EH, Leaver-Fay A, Baker D. Role of conformational sampling in computing mutation-induced changes in protein structure and stability. Proteins. 2011;79: 830–838. doi:10.1002/prot.22921

17. Choe H-W, Kim YJ, Park JH, Morizumi T, Pai EF, Krauss N, et al. Crystal structure of metarhodopsin II. Nature. 2011;471: 651–655. doi:10.1038/nature09789

18. Nguyen ED, Norn C, Frimurer TM, Meiler J. Assessment and Challenges of Ligand Docking into Comparative Models of G-Protein Coupled Receptors. PLoS ONE. 2013;8: e67302. doi:10.1371/journal.pone.0067302
