# Supplementary figures and images for: An Integrated Framework Advancing Membrane Protein Modeling and Design
Source: PLoS Comput Biol. 2015 Sep 1;11(9):e1004398. doi: 10.1371/journal.pcbi.1004398 (PMC4556676; doi:10.1371/journal.pcbi.1004398)

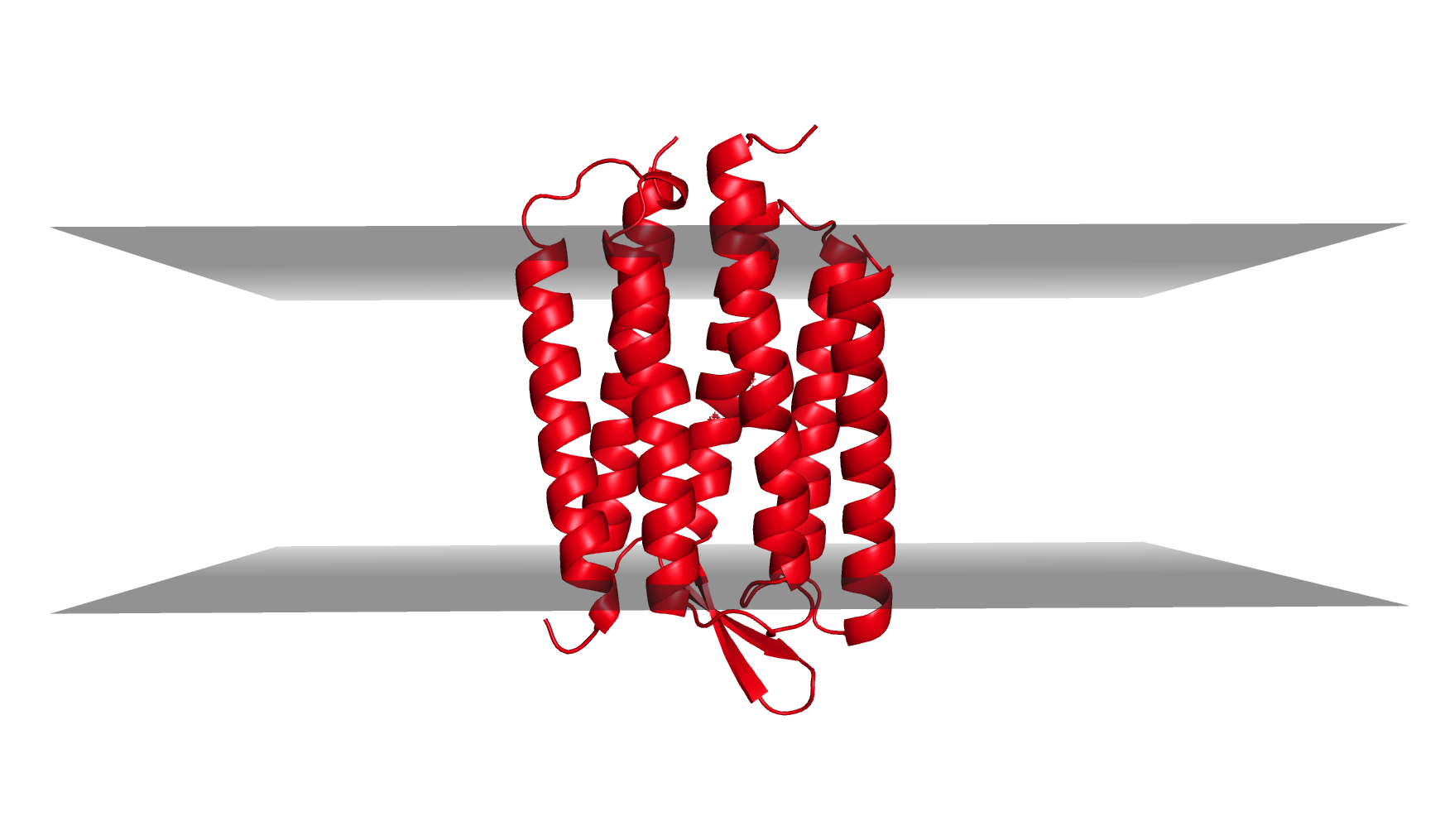

Supplement: S6 File — This protocol capture contains the steps, input files, and example output files necessary to run the MPPyMOLViewer protocol described in this manuscript. For simplification, we only describe visualization of bacteriorhodopsin these files. The supplementary files are included with the Rosetta3 software suite under the directory Rosetta/demos/protocol_capture/MPPyMOLViewer. (GZ) [file pcbi.1004398.s006.gz › MPPyMOLViewer/example_outputs/1c3w.png]
